# Protection from DNA re-methylation by transcription factors in primordial germ cells and pre-implantation embryos can explain trans-generational epigenetic inheritance

**DOI:** 10.1186/s13059-020-02036-w

**Published:** 2020-05-18

**Authors:** Isaac Kremsky, Victor G. Corces

**Affiliations:** Department of Human Genetics, Emory University School of Medicine, Atlanta, GA 30322 USA

**Keywords:** Chromatin, Transcription, DNA methylation, Spermatogenesis, Oogenesis, Fertilization, Embryo, Development, Trans-generational inheritance

## Abstract

**Background:**

A growing body of evidence suggests that certain epiphenotypes can be passed across generations via both the male and female germlines of mammals. These observations have been difficult to explain owing to a global loss of the majority of known epigenetic marks present in parental chromosomes during primordial germ cell development and after fertilization.

**Results:**

By integrating previously published BS-seq, DNase-seq, ATAC-seq, and RNA-seq data collected during multiple stages of primordial germ cell and pre-implantation development, we find that the methylation status of the majority of CpGs genome-wide is restored after global de-methylation, despite the fact that global CpG methylation drops to 10% in primordial germ cells and 20% in the inner cell mass of the blastocyst. We estimate the proportion of such CpGs with preserved methylation status to be 78%. Further, we find that CpGs at sites bound by transcription factors during the global re-methylation phases of germline and embryonic development remain hypomethylated across all developmental stages observed. On the other hand, CpGs at sites not bound by transcription factors during the global re-methylation phase have high methylation levels prior to global de-methylation, become de-methylated during global de-methylation, and then become re-methylated.

**Conclusions:**

The results suggest that transcription factors can act as carriers of epigenetic information during germ cell and pre-implantation development by ensuring that the methylation status of CpGs is maintained. These findings provide the basis for a mechanistic description of trans-generational inheritance of epigenetic information in mammals.

## Background

Evidence for the transmission of phenotypic traits via inter- and trans-generational epigenetic inheritance in mammals has grown substantially in recent years. However, the underlying mechanisms responsible for these phenomena have remained elusive [1, 2]. DNA methylation is arguably the best candidate carrier of epigenetic information and an appealing option to explain inter- and trans-generational epigenetic inheritance, since it is heritable across rounds of DNA replication. However, the genome is globally de-methylated in mammals at two developmental stages—primordial germ cells (PGCs) and pre-implantation embryos. Global DNA methylation levels fall below 10% in PGCs [3] and below 20% in the inner cell mass (ICM) stage of pre-implantation development [4]. Maintenance of CpG methylation during PGC and pre-implantation development at endogenous retroviral elements of the Intracisternal A Particle (IAP) type are known to be involved in some cases of trans-generationally inherited epialleles, including the *Agouti viable yellow* and *Axin-Fused* loci [5–8]. In these examples, the phenotype is correlated with the DNA methylation status of the IAP inserted near the relevant genes, driving their expression via an alternate promoter within the IAP long terminal repeat (LTR) [7, 9]. CpGs at IAPs are known to be resistant to global de-methylation during PGC development [3]; thus, maintenance of CpG methylation status at these loci across PGC and pre-implantation development is the likely mechanism of epigenetic inheritance.

While it is possible that the remaining 10% of methylated CpGs during PGC development is sufficient to explain inter- and trans-generational epigenetic inheritance of all known heritable epiphenotypes, genome-wide BS-seq studies show that the methylation status of a substantial proportion of CpG sites is faithfully recapitulated before and after global de-methylation and re-methylation across both PGC [3] and embryonic [4] development. That is, a substantial number of CpGs that are methylated prior to global de-methylation are first de-methylated and then re-methylated, whereas many CpGs that are not methylated prior to global de-methylation remain unmethylated even after global re-methylation. This suggests that additional carrier(s) of epigenetic information must exist to maintain the memory of CpG methylated states after reprogramming events. Identifying the additional carrier(s) is essential for understanding mechanisms of inter- and trans-generational epigenetic inheritance. The extent to which CpG methylation status is faithfully maintained has not been previously quantified.

A number of candidate carriers of epigenetic information besides DNA methylation have been proposed to be involved in mechanisms of inter- and trans-generational epigenetic inheritance, including miRNAs, piRNAs, and tRNAs [2, 10–13]. However, mechanisms explaining these phenomena are generally thought to require that epigenetic information is passed between different types of molecular carriers over the course of development [14]. Although alterations in sperm non-coding RNAs (ncRNAs) have been shown to induce trans-generational effects on phenotypes [10, 11, 13], evidence is lacking that the alterations in sperm ncRNAs persist beyond the F_1_ generation. On the contrary, mice subjected to stress have altered miRNAs in the F_1_ that induce a trans-generational change in phenotype, but those miRNAs return to normal levels in F_2_ sperm, despite the fact that F_3_ mice still displayed the same alterations in phenotype that were observed in the F_1_ and F_2_ generations due to stress in the F_0_ [10]. This suggests that alterations in miRNAs are capable of inducing a trans-generational effect on phenotype, but that some other molecular carrier may be responsible for the trans-generational maintenance of the altered phenotype [10].

Histone modifications are widely studied molecular carriers of epigenetic information. Studies have found that H3K4me3 and H3K27me3 can be passed on from oocytes to pre-implantation embryos, but the same studies found that these marks are globally reprogrammed on paternal alleles upon fertilization [15, 16]. To date, no studies have identified a histone modification present in sperm that is maintained upon fertilization. Therefore, while histone modifications could act in mechanisms of maternal inter- and trans-generational epigenetic inheritance, they do not appear to be sufficient to explain paternal mechanisms. Nucleosomes containing H2A.Z and H3K4me1 have been shown to antagonize de novo CpG methylation in zebrafish during pre-implantation development [17], whereas in mammals, methylation of H3K4 has been shown to antagonize de novo methylation due to preferential binding of Dnmt3l to unmethylated H3K4 [18]. However, this mechanism does not confer complete resistance to de novo methylation. Thus, the precise molecular carriers involved in trans-generational maintenance of epialleles have to this point still not been identified.

Transcription factors (TFs) could also serve as carriers of epigenetic information, but their possible role in this process has received only moderate attention. TFs are present on the genomes of mature gametes [19, 20], and there is evidence that TF binding can influence DNA methylation at the binding site, both by direct steric hindrance of DNA methyltransferases and by recruitment of Tet enzymes to specific TFs bound to DNA [21]. In addition, a class of TFs known as pioneer factors can bind to nucleosomes and can stabilize nucleosome positioning [22], suggesting that TFs could also be involved in the placement of marker nucleosomes to inhibit de novo DNA methylation. TFs thus represent a plausible candidate that could pass on epigenetic information when DNA methylation is erased, acting as mediators that can allow cells to preserve DNA methylation patterns across PGC differentiation and pre-implantation development.

In order to test the hypothesis that TFs might act as mediators of epigenetic memory during global DNA methylation loss, we integrated publicly available BS-seq [3, 4], RNA-seq [3, 15], DNase-seq [23, 24], and ATAC-seq [20, 25, 26] data across multiple stages of PGC and embryonic development. We find a striking correlation between the presence or absence of bound TFs during the global re-methylation phase of PGCs and embryonic stem cells (ESCs), and the methylation status of CpGs at their core binding sequence in sperm, epiblasts, and adult somatic tissues. The results suggest that TFs may be involved in the maintenance of epigenetic information, ensuring that the methylation state of the majority of CpGs is preserved trans-generationally. This TF-mediation model of epigenetic inheritance leads to testable predictions for how both inter- and trans-generational epigenetic inheritance might occur mechanistically.

## Results

### TF binding at CpGs in E14.5 male PGCs predicts DNA methylation patterns throughout male PGC development

PGCs first appear in the epiblast at E6.5 of mouse embryonic development, and in male embryos, they eventually become sperm. As PGCs migrate to the genital ridge and differentiate, their genomes become de-methylated, and de-methylation is completed by E13.5, with 10% of CpGs in the genome still remaining methylated [3], mostly at transposable element sequences. After this time, male PGCs commence the global re-methylation phase, and average levels of methylation reach 50% by E16.5 [3] and nearly 80% in the mature sperm [4]. The paternal genome is largely de-methylated immediately after fertilization whereas the maternal genome is de-methylated more slowly during pre-implantation development. Both genomes retain an average of 20% of CpG methylation in the ICM of the blastocyst at E3.5 [4]. Re-methylation of the paternal and maternal chromosomes takes place during subsequent embryonic development, and ~ 70% of CpGs are methylated in the epiblast at E6.5 [3].

**Fig. 1:**
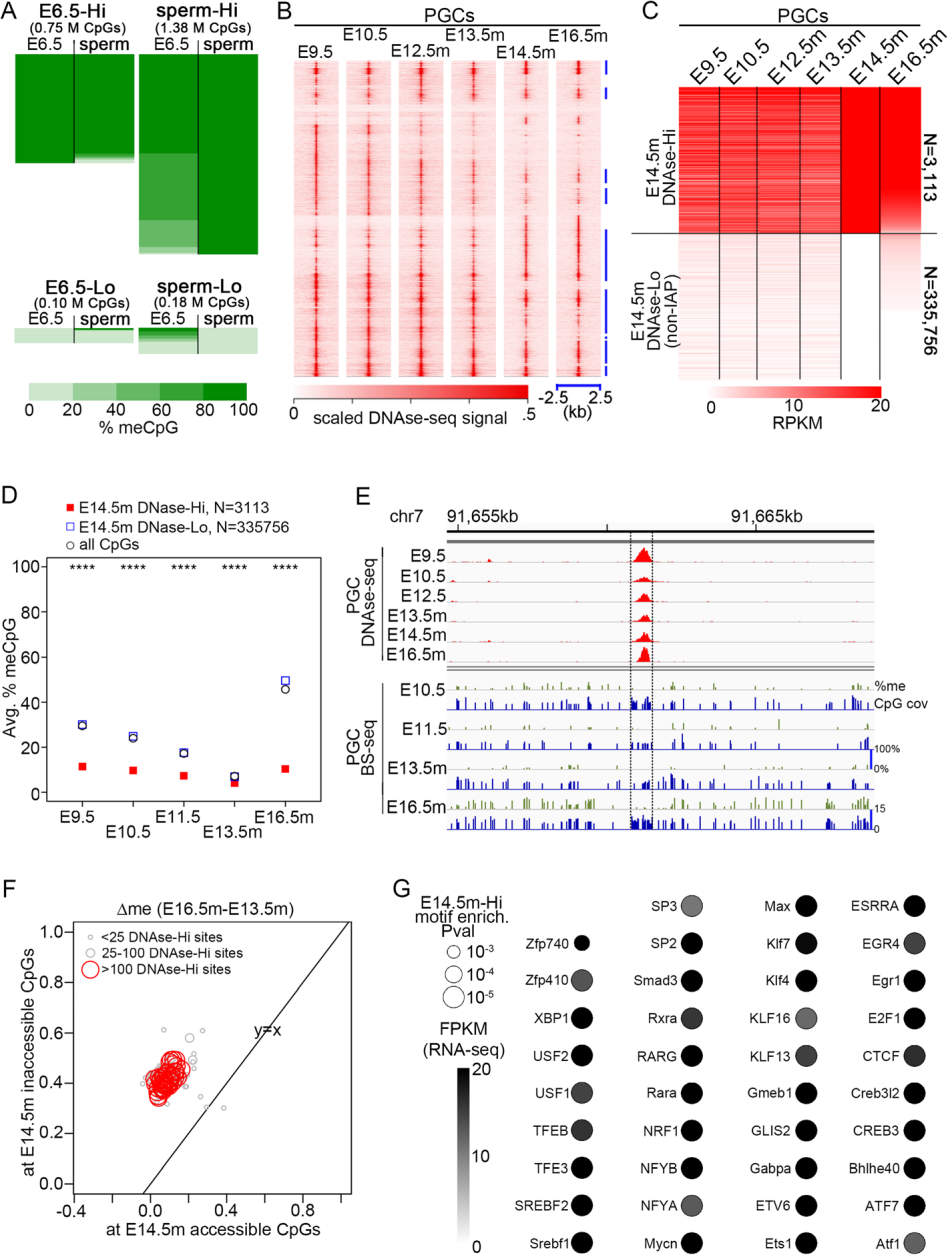
TF binding at CpGs in E14.5m PGCs predicts DNA methylation patterns throughout male PGC development. **a** Heatmaps of % methylation from BS-seq signal in E6.5 epiblasts and sperm. Each row represents a CpG with at least 10 BS-seq reads in both samples. The heatmaps are divided as follows: CpGs with very high methylation (> 80%) in epiblast (top left), CpGs with very low methylation (< 20%) in epiblasts (bottom left), CpGs with very high methylation in sperm (top right), and CpGs with very low methylation in sperm (bottom right). Heatmap heights are scaled to the total number of CpGs. **b** DNase-seq signal centered at binding sequences called by fimo that overlap a peak summit in at least one of the samples displayed, ordered by hierarchical clustering. Vertical blue lines at the right show regions of high DNAse signal across PGC development. **c** RPKM values within binding sequences called by fimo, separated into those that have high DNase signal in E14.5m (DNase-Hi, RPKM > 20) and those that have RPKM = 0 (DNase-Lo). **d** Average methylation of CpGs within E14.5m DNase-Hi and DNase-Lo regions, weighted by the number of BS-seq reads at each CpG. The numbers displayed correspond to the number of TF binding sequences at which CpG methylation was averaged. *P* values are by Fisher’s exact test. *P* value cutoffs (E14.5 DNase-Hi vs. E14.5 DNase-Lo) *P < 0.01; **P < 10^−3^; ***P < 10^−5^; ****P < 10^−10^. **e** Region with DNase accessibility and low local DNA methylation throughout PGC development. CpG cov indicates the BS-seq read coverage. **f** Changes in DNA methylation between E14.5m and E16.5m PGCs. Each point represents the average DNA methylation change (E16.5m minus E13.5m PGCs) at CpGs overlapping a binding sequence for a specific TF. The *x*-axis gives the average value for E14.5m PGC DNase-Hi sites, while the *y*-axis gives the average value for DNase-Lo sites. **g** Significantly enriched motifs at E14.5m DNase-Hi sites. *P* values are by Fisher’s exact test

We sought to quantify the degree to which the methylation status of DNA is preserved across PGC and pre-implantation development by comparing the CpG methylation state of E6.5 epiblast cells with that of sperm, with the caveats that only a subset of epiblast cells give rise to the germline and that epiblast cells have not yet undergone complete re-methylation. Using previously published genome-wide bisulfate sequencing (BS-seq) data [3, 4], we find that 91% of CpGs with very high (> 80%) methylation in the epiblast also have the same methylation level in sperm, whereas 90% of CpGs that have very high methylation in sperm have > 50% methylation in epiblast cells (Fig. 1a, top). Similarly, of the CpGs with very low (< 20%) methylation in epiblasts, 83% also have very low methylation in sperm, whereas 47% of CpGs with very low methylation in sperm also have very low methylation in epiblasts (Fig. 1a, bottom). Given that epiblasts are still undergoing global de novo methylation whereas sperm are fully methylated, the methylation levels in sperm are more reliable to estimate the proportion of CpGs whose methylation status is faithfully maintained after global de-/re-methylation. Although BS-seq data in successive generations of sperm are optimal to precisely determine the amount of CpGs with preserved methylation status across generations, comparing the epiblast data to sperm can give us a reasonable estimate of the amount of preserved CpGs. Of the high confidence CpGs in sperm, only 9% had a methylation level in between 80 and 20%, i.e., roughly 91% of CpGs in sperm have either > 80% or < 20% methylation. Conservatively, assuming that the 9% of intermediately methylated CpGs in sperm are not preserved across generations, we estimate (see the “Methods” section) that the methylation level of 78% of CpGs is preserved before and after the global de-methylation and re-methylation phases of PGC and pre-implantation development and, therefore, across generations.

The mechanisms by which PGCs retain a memory of the previous DNA methylation state after global DNA de-methylation are unclear. We thus sought to test the hypothesis that the presence of DNA-bound TFs precludes re-methylation of bound sequences. To this end, we used publicly available DNase-seq data obtained in PGCs at different stages of differentiation, from days 9.5–13.5 of embryonic development (E9.5–E13.5), when DNA de-methylation is completed, to E14.5–E16.5, which coincide with the re-methylation of male PGCs [23]. We analyzed the pattern of TF dynamics at distal CpG sites (> 2.5 kb from annotated TSSs at sites presumed to be enhancers) using unsupervised clustering and found a number of distinct temporal patterns of TF occupancy (Fig. 1b). Some TF binding sites remain accessible throughout PGC development, whereas others either gain or lose accessibility at specific stages. However, the accessibility remains largely fixed between embryonic days E14.5 and E16.5 of male PGC development (E14.5m and E16.5m, respectively) (Fig. 1b).

To determine the relationship between TF occupancy and DNA methylation during the global re-methylation phase, we selected TF binding sequences with high DNase-seq signal in E14.5m PGCs (DNase-Hi) and compared them to a control set of TF binding sites with no DNase-seq signal in E14.5m (DNase-Lo) (Fig. 1c). To identify DNase-Lo sites, we obtained DNase-seq peaks for all available mouse tissues from ENCODE [27], including all peaks from the PSU Hardison [28] and UW Stamatoyannopoulous [29–31] labs. We merged all ENCODE peaks with all peaks from PGCs and ESCs, and we used fimo [32] to identify peaks containing known TF binding sequences. The default set of both human and mouse TF motifs were used to scan for TF binding sequences, since the set of mouse motifs is smaller than that of human, and thus would allow us to infer a greater number of likely TFs bound at DNAse-Hi sites. DNase-Hi and DNase-Lo sites consist only of the merged set of binding sequences themselves, and not the rest of the peak regions, since those are the presumed binding sites of the TFs.

The level of DNase-seq signal at DNase-Hi and DNase-Lo sites is largely unchanged between E14.5m and E16.5m but is distinct at all other PGC stages (Fig. 1c; Additional file 1: Figure S1A). The dynamic changes in accessibility in the early mitotically active PGC stages are consistent with dynamic changes in DNAse-seq accessibility previously observed at distal TF binding sites, likely due to the loss of trans-acting factor affinity for chromatin [33]. Thus, in many cases, it is possible that E14.5m DNAse-Hi sites are still bound by core TFs in early PGCs but have low DNAse-seq accessibility due to a loss of co-factor binding. On the other hand, the close similarity in mitotically arrested PGCs between E14.5m and E16.5m suggests that these TF binding sites remain persistently bound during the re-methylation phase of PGC development. It has been shown using ATAC-seq that regulatory elements can remain persistently accessible during dynamic changes in development [34]. Therefore, we cannot rule out the possibility that specific TFs bound at each site change dynamically during the DNA re-methylation phase. Nonetheless, these data suggest the persistent presence of bound TFs at E14.5m DNAse-Hi sites during the global re-methylation phase from E14.5 to E16.5 in male PGCs.

We then compared the average DNA methylation levels at DNase-Hi and DNase-Lo CpGs across PGC development (Fig. 1d, e; Additional file 1: Figure S1B, C). The DNA methylation pattern at the DNase-Lo sites resembles that of the global average: they are progressively de-methylated from E9.5 to E13.5m, and re-methylated thereafter. On the other hand, DNase-Hi sites remain largely unmethylated at all stages of PGC development for which data exist, and methylation levels at the DNase-Hi sites are significantly lower than those at DNase-Lo sites at all PGC stages. We then examined the average change in methylation from E13.5m to E16.5m at TF binding sites for each TF separately, limiting the set of TFs to only those that have RNA-seq signal at E16.5m. For every such TF binding sequence, sites with high DNase-seq signal have a smaller increase in DNA methylation levels than sites that are not bound by TFs (Fig. 1f). A motif enrichment analysis revealed 39 significantly enriched TFs that are expressed in E16.5m PGCs (Fig. 1g; Additional file 1: Figure S1D; Additional file 2: Table S1). The majority of these TFs have clear DNase footprints at their DNase-Hi sites (Additional file 1: Figure S2), suggesting that TFs are bound specifically at the majority of DNase-Hi binding sequences identified in E14.5m.

CpG islands remain predominantly unmethylated in a cell type-independent manner. We therefore examined the CpG density at the DNAse-Hi sites and found it to be significantly lower than the CpG density of DNAse-Lo sites overlapping CpG islands (Additional file 1: Figure S4A) and more similar to the CpG density at DNAse-Lo sites not overlapping CpG islands (Additional file 1: Figure S4B). This suggests that the majority of E14.5m DNAse-Hi sites do not occur at CpG islands. These sites tend to be within or in close proximity to gene bodies, although DNAse-Hi sites can be found as far as 2 megabases away from the nearest gene (Additional file 1: Figure S4C).

Taken together, these results support the hypothesis that TFs can act as mediators of epigenetic information during the PGC global re-methylation phase by determining subsequent DNA methylation levels. CpGs bound by TFs at E14.5m tend to be unmethylated throughout PGC development, whereas CpGs that do not bind a TF at E14.5m are methylated early in PGC development, become progressively de-methylated until E13.5, and then become re-methylated in males. Females do not undergo re-methylation until much later than E13.5, and thus, we were unable to test this phenomenon in females.

### Global TF binding and DNA methylation patterns during the male PGC and ESC re-methylation phases are highly correlated

**Fig. 2:**
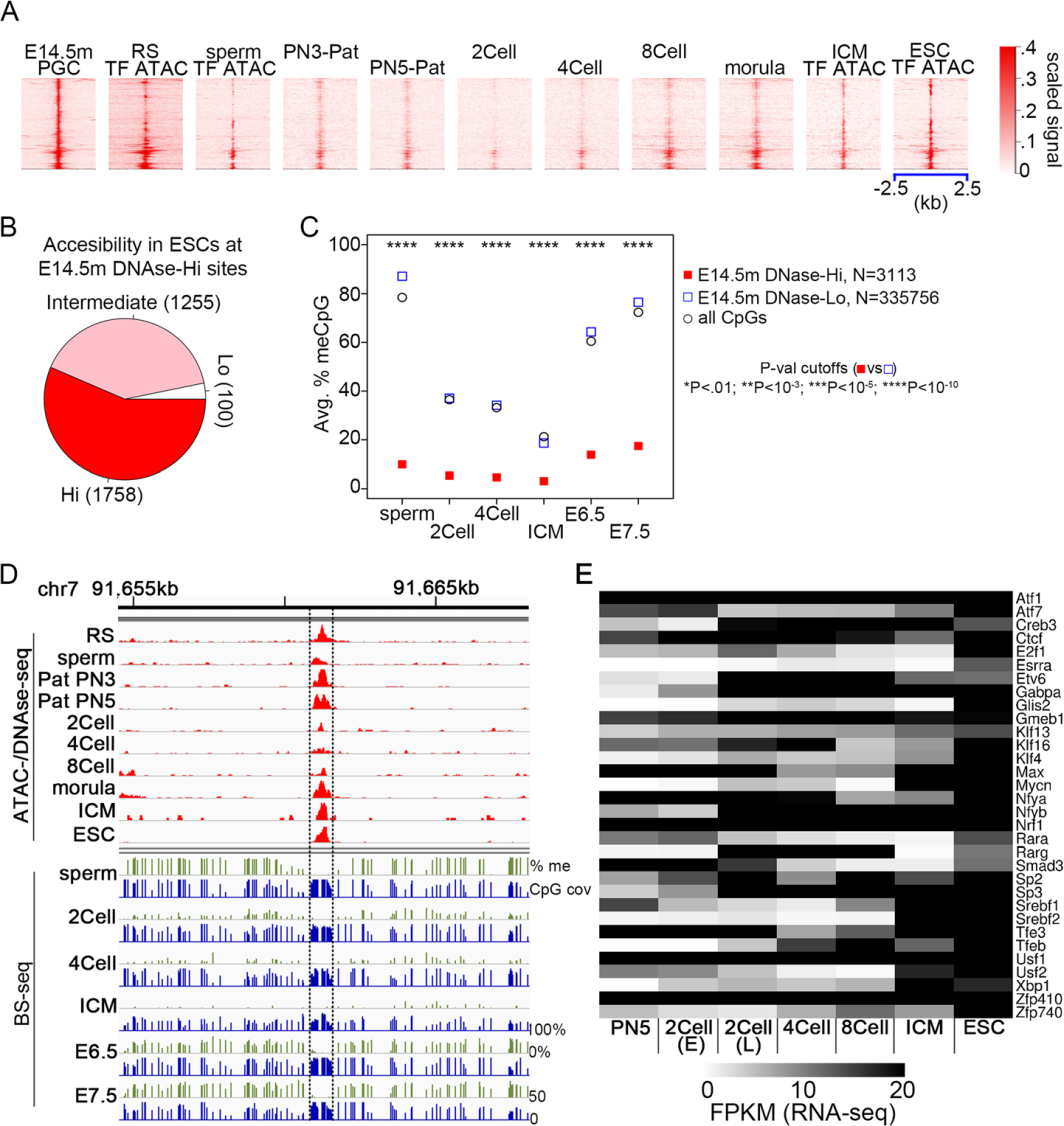
Global TF binding and CpG methylation patterns during male PGC and ESC differentiation are highly correlated. For all cases where ATAC-seq signal was used in this figure, only ATAC-seq fragments < 115 bp, which indicate TF binding, were used. **a** Heatmap at the indicated stages of gamete and embryonic development, centered at E14.5m PGC DNase-Hi sites ordered by hierarchical clustering. RS, round spermatids. PN3_P, PN5_P, Paternal PN3 and PN5 pronucleus, respectively. Data from ATAC-seq are labeled as such; all other data are from DNAse-seq. **b** Pie chart showing the proportion of E14.5m PGC DNase-Hi sites with high, intermediate, or low ATAC-seq signal in ESCs. **c** Average methylation of CpGs within E14.5m DNase-Hi and DNase-Lo regions, weighted by the number of BS-seq reads at each CpG. The numbers displayed correspond to the number of TF binding sequences at which CpG methylation was averaged. *P* values are by Fisher’s exact test. *P* value cutoffs (E14.5 DNase-Hi vs. E14.5 DNase-Lo) *P < 0.01; **P < 10^−3^; ***P < 10^−5^; ****P < 10^−10^. **d** Example displaying DNase-seq and BS-seq signal with peaks in the same region as in Fig. 1e. **e** Heatmap of RNA-seq signal at the indicated stages for the significantly enriched TFs at E14.5m PGC DNase-Hi sites from Fig. 1g

The fact that approximately 78% of CpGs preserve their methylation status between sperm and E6.5 epiblasts (Fig. 1a) suggests that the methylation status is preserved not just across global de-methylation followed by re-methylation of PGCs, but also across global de- and re-methylation in pre-implantation embryos. We therefore wondered if the same pattern of TF binding during the global re-methylation phase of PGCs would also be present during the re-methylation of ESCs. To test this, we examined ATAC-seq subnucleosomal-size reads in ESCs [25] at the E14.5m PGC DNase-Hi sites. We found that the vast majority of E14.5m DNase-Hi sites have either intermediate or high ATAC-seq signal in ESCs (Fig. 2a, b). Less than 5% have low ATAC-seq signal in ESCs (Fig. 2b).

We next examined the DNA methylation levels throughout pre-implantation development at E14.5m PGC DNase-Hi and DNase-Lo sites using published BS-seq data [4]. We found the same behavior as in PGCs: DNase-Lo sites follow the global average pattern of de-methylation after fertilization and up to the ICM stage, followed by re-methylation thereafter, whereas DNase-Hi sites remain lowly methylated at all stages for which data exist (Fig. 2c, d; Additional file 1: Figure S4A-C). Binding sequences for the same set of 39 TFs identified above (Fig. 1g) show less change in DNA methylation levels between ICM and E7.5 embryos when bound by a TF than when unbound (Additional file 1: Figure S4A). As is the case in PGCs, most of these TFs show clear footprints at their binding sequences in ESCs (Additional file 1: Figure S5). Each of these 39 TFs is expressed in ESCs based on RNA-seq data (Fig. 2e).

Taken together, these results, along with those discussed in the previous section, suggest that despite two rounds of global de-methylation followed by re-methylation, the methylation status of the majority of CpGs in the mammalian genome is preserved across embryonic development, and that the binding of TFs during the global re-methylation phases is associated with maintenance of hypomethylation across development. On the other hand, unbound TF binding sites are methylated both before and after the two rounds of global de-/re-methylation.

### Bound TFs co-localize with co-factors capable of resisting de novo methylation

Our results thus far demonstrate a striking correlation between the presence of TFs and maintenance of a hypomethylated state, but do not address whether the role of TFs is direct or indirect. We therefore examined whether bound TF sites contain any co-factors known to affect DNA methylation. It has been shown that H3K4me3 represses de novo DNA methylation because Dnmt3l binds exclusively to unmethylated H3K4 [18]. Although H3K4me3 is typically found at promoters rather than enhancers, it has been shown that distal H3K4me3 domains associated with hypomethylated DNA are critical in the transition from oocytes to zygotes [35]. In addition, “orphan” bivalent domains containing both H3K4me3 and H3K27me3 at regions distal to promoters have been observed in E11.5 PGCs [36]. Finally, Tet enzymes are often associated with bound TFs [37]. We therefore examined ChIP-seq data obtained in ESC for H3K4me3 and H3K27me3 [15] as well as Mll2 and Ash2L [38], factors involved in the methylation of H3K4. We also examined ChIP-seq of Tet1 at these regions in ESCs [39], and Dnmt3a1 [39] as a negative control. We observe enrichment for Mll2, Ash2l, and Tet1 at virtually all E14.5m PGC DNAse-Hi sites (Additional file 1: Figure S6A-C). Consistent with this, nearly all of these sites are flanked by H3K4me3-containning nucleosomes and are depleted of Dnmt3a1 (Additional file 1: Figure S6A-C). H3K27me3 was also present around many of these sites (Additional file 1: Figure S6A), but the signal is weaker than H3K4me3, suggesting a more critical role for H3K4me3 at these sites, consistent with its known function of actively blocking de novo DNA methylation. The enrichment for these factors does not occur at E14.5m PGC DNAse-Lo sites (Additional file 1: Figure S6B), suggesting that TF binding is required for the recruitment of these co-factors.

In order to test whether TF binding is a cause or consequence of co-regulator activity, we looked at both TF ATAC-seq signal and H3K4me3 ChIP-seq signal in ICM and ESCs [15] at DNAse-Hi sites from E14.5m PGC. The majority of sites show enrichment for TF ATAC-seq signal in both ICMs and ESCs, whereas most are not enriched in H3K4me3 signal until ESCs (Additional file 1: Figure S6D,E). This suggests that TF binding precedes the placement of H3K4me3, consistent with a causal role of TF binding in the recruitment of H3K4me3, not vice versa. We also looked at the enrichment of H3K4me3 and H3K27me3 ChIP-seq in E11.5 PGCs [36] as well as in epiblast-like cells and PGC-like cells [40] at E14.5m DNAse-Hi sites. Enrichment of H3K4me3 was observed at only a subset of sites for all of the PGC-like and epiblast-like data (Additional file 1: Figure S6F). However, the same pattern occurred in the ESCs from which these cells were derived (Additional file 1: Figure S6F), whereas virtually all of these sites are flanked by H3K4me3 in the other ESC dataset (Additional file 1: Figure S6A). This suggests that the ESC lines from which the epiblast-like and PGC-like cells were derived may lack TF binding at many of the distal sites which are normally occupied in vivo. Consistent with this, nearly all sites were flanked by H3K4me3 in E11.5 PGCs in vivo (Additional file 1: Figure S6F). Nonetheless, the late-stage day 6 PGC-like cells show stronger H3K4me3 signal than the early-stage day 2 PGC-like cells. Given the similar global DNA methylation reprogramming patterns that occur in PGCs and pre-implantation embryos, it is likely that this distal H3K4me3 is lost during global DNA de-methylation and is re-established after the binding of TFs during the re-methylation phase of PGCs and ESCs. Consistent with this, expression levels of Mll2 (Kmt2d) from RNA-seq data diminish from the 2Cell to the 8Cell stage but become progressively higher from the ICM to ESCs (Additional file 1: Figure S6G). Similarly, in E11.5 PGCs, Mll2 is not expressed, but it is expressed in E13.5m and E16.5m PGCs (Additional file 1: Figure S6H).

### CpG methylation reprogramming across PGC development is associated with the binding of a specific class of reprogramming TFs

**Fig. 3:**
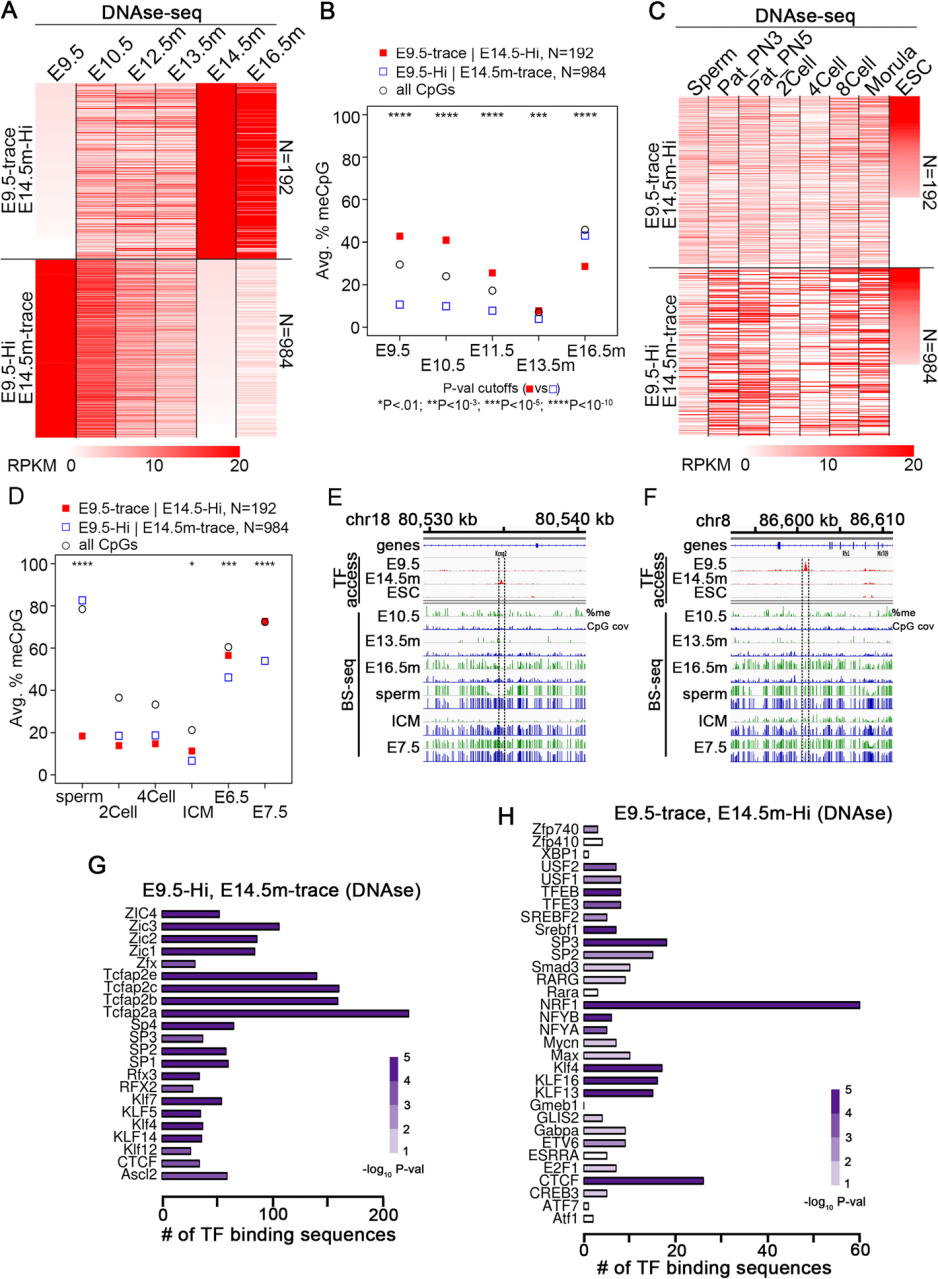
CpG methylation is reprogrammed only at a small fraction of TF binding sites, at binding sites for specific reprogramming TFs during male PGC and ESC development. **a** Heatmaps of DNase-seq signal at the indicated sites during PGC development. E9.5-trace, E14.5m-Hi sites are those that have FPKM < 1 in E9.5 PGCs and FPKM > 20 in E14.5m PGCs, while E9.5-Hi, E14.5m-trace sites have FPKM > 20 in E9.5 PGCs and FPKM < 1 in E14.5m PGCs. The two heatmaps are not drawn to scale in order to enable better viewing of both sets. **b** Average DNA methylation levels of CpGs at the indicated regions and stages of PGC development, weighted by the number of BS-seq reads at each CpG. The numbers displayed correspond to the number of TF binding sequences at which CpG methylation was averaged. *P* values are by Fisher’s exact test. *P* value cutoffs (E9.5-trace, E14.5m-Hi vs. E9.5-Hi, E14.5m-trace) *P < 0.01; **P < 10^−3^; ***P < 10^−5^; ****P < 10^−10^. **c** Heatmaps of DNase-seq signal at the indicated sites during gamete and pre-implantation development. E9.5-trace, E14.5m-Hi sites are those that have FPKM < 1 in E9.5 PGCs and FPKM > 20 in E14.5m PGCs, while E9.5-Hi, E14.5m-trace sites have FPKM > 20 in E9.5 PGCs and FPKM < 1 in E14.5m PGCs. The two heatmaps are not drawn to scale in order to enable better viewing of both sets. **d** Average DNA methylation levels of CpGs at the indicated regions and stages of pre-implantation development, weighted by the number of BS-seq reads at each CpG. The numbers displayed correspond to the number of TF binding sequences at which CpG methylation was averaged. *P* values are by Fisher’s exact test. *P* value cutoffs (E9.5-trace, E14.5m-Hi vs. E9.5-Hi, E14.5m-trace) *P < 0.01; **P < 10^−3^; ***P < 10^−5^; ****P < 10^−10^. **e** Example showing the DNA methylation across development at an E9.5-trace, E14.5m-Hi site with a CTCF binding sequence. **f** Example showing the DNA methylation across development at an E9.5-Hi, E14.5m-trace site with a CTCF binding sequence. CpG cov indicates the BS-seq read coverage. **g**, **h** Bar plots showing the number of motif hits for each TF at the indicated set of regions. Statistical significance of the enrichment of each TF motif, based on Fisher’s exact test, is indicated by the color purple

Given that the majority of CpGs have a preserved methylation status across embryonic development, we sought to characterize the CpGs whose methylation status is not preserved. We therefore define “reprogramming” here not as a loss in methylation during the global de-methylation phases of PGCs and pre-implantation embryos, but rather a break in the pattern of either (1) being methylated in the epiblast, sperm, and adult somatic tissue despite global de-methylation happening in PGCs and pre-implantation embryos, or (2) remaining hypomethylated across development, including in the epiblast, sperm, and adult somatic tissue. Under this definition, a “reprogramming TF” is one that binds to a methylated region of DNA and reprograms it as defined above. There is evidence that some TFs, including CTCF and Esrrb, can bind to methylated DNA and are associated with de-methylation of their binding sites and the surrounding regions [41–44]. We thus wondered whether this is also the case during PGC development. To test this, we took the set of regions with low DNase-seq signal in E9.5 PGCs but high DNase-seq signal in E14.5m PGCs (E9.5-trace, E14.5m-Hi) and compared them to regions with high signal at E9.5 PGCs and low signal at E14.5m PGCs (E9.5-Hi, E14.5m-trace). Both TF binding and DNA methylation were compared between these sites throughout germline and embryonic development (Fig. 3a–f). We found that the DNA methylation signal was reprogrammed according to its change in DNase-seq signal: E9.5-Hi, E14.5m-trace sites have low DNA methylation at E9.5 but high DNA methylation at E16.5m, whereas E9.5-trace, E14.5m-Hi sites have high DNA methylation levels at E9.5 and low levels at E16.5m (Fig. 3a, b).

TF binding in sperm and pre-implantation embryos occurs at some but not all of the E9.5-Hi, E14.5m-trace and E9.5-trace, E14.5m-Hi sites, and specific sites gain or lose TF occupancy at different stages in this time range (Fig. 3c). However, the DNA methylation levels of E9.5-Hi, E14.5m-trace sites remain high in sperm (Fig. 3d), consistent with their high levels in E16.5m PGCs. They then undergo a drastic drop in methylation after fertilization and are only methylated at intermediate levels in the epiblast stage (Fig. 3d). The change from ~ 80 to ~ 20% methylation from sperm to 2Cell at E9.5-Hi, E14.5m-trace sites can be partially accounted for by the fact that these sites are only methylated at ~ 50% in oocytes (Additional file 1: Figure S7A); however, E9.5-Hi, E14.5m-trace site methylation is still at ~ 65% when averaging between sperm and oocytes and drops to ~ 20% at the 2Cell stage, indicating a relatively rapid de-methylation after fertilization. Since they are hypomethylated in E9.5 PGCs but not in E6.5 embryos, this suggest the DNA methylation at many of these sites is reprogrammed sometime between the epiblast and early PGC stages, hinting at a possible role for these sites in early PGC development. On the other hand, E9.5-trace, E14.5m-Hi sites have a TF bound during the PGC re-methylation phase whereas many of them are not bound in ESCs. Therefore, the reprogramming in this case may simply be due to the uniqueness of many of these binding sites to the PGC re-methylation phase. On the other hand, there are slightly lower DNA methylation levels in sperm than in E16.5m PGCs, and thus, it is also possible that reprogramming occurs throughout sperm development at many sites (Fig. 3d).

To determine the nature of the TFs that may be involved in these reprogramming events, we performed a motif enrichment analysis (Fig. 3g, h). The putative reprogramming TFs that are expressed in E16.5m PGCs (Fig. 3h) include CTCF, Esrra, and Nrf1. Esrra is known to be involved in the facultative activation of autophagy genes via binding to their promoters [45], and it shares homology with Esrrb, which, as mentioned, has been found to bind methylated DNA and promote de-methylation of adjacent nucleotides [46]. Although Nrf1 has been shown to be unable to bind methylated DNA, Nrf1 binding is often concurrent with the binding of other TFs nearby, such as CTCF, some of which can promote its interaction with DNA by promoting de-methylation of the Nrf1 binding site [47]. The fact that Nrf1 is the most highly enriched TF may reflect the need to be adjacent to a reprogramming TF binding site. Of the putative E9.5-Hi, E14.5m-trace TFs (Fig. 3h), Klf5, Rfx2, Rfx3, Tcfap2c, Zfx, and Zic3 are expressed in E11.5 PGCs, but their expression is diminished or eliminated in E16.5m PGCs (Additional file 1: Figure S7B), consistent with binding in E11.5 that is lost in E14.5m and thus making these promising candidate E9.5-Hi, E14.5m-trace TFs.

H3K4me3 can be found in ESCs and E11.5 PGCs at many of the E9.5-trace, E14.5m-Hi and E9.5-Hi, E14.5m-trace sites (Additional file 1: Figure S7C). In addition, a subset of these are present in a poised enhancer-like state, with enrichment for H3K27me3 (Additional file 1: Figure S7C). Interestingly, the enrichment pattern of H3K4me3 is conserved between ESCs and E11.5 PGCs, whereas H3K27me3 shows distinct patterns of enrichment at each of these stages, suggesting that these regions may undergo dynamic phases of poising and activation during embryonic development. There are 105 genes expressed in E11.5 PGCs within 10 kb of E9.5-Hi, E14.5m-trace sites, and similarly, there are 12 genes expressed in E16.5m PGCs within 10 kb of E9.5-trace, E14.5m-Hi sites (Additional file 3). We input these genes into an Enrichr pathway enrichment analysis [48, 49]; the top GO Biological Process for genes associated with E9.5-trace, E14.m-Hi sites was “positive regulation of mitochondrial calcium ion concentration”, while the top for E9.5-Hi, E14.5m-Hi associated genes was “regulation of cell cycle G1/S phase transition” (Additional file 1: Figure S7D).

Taken together, these results suggest that CpGs at binding sites for a class of TFs can become reprogrammed at specific stages of embryonic development. The TFs shown in Fig. 3g, h therefore represent putative reprogramming TFs that are either capable of binding methylated DNA and promoting its de-methylation, or generally binding adjacent to a TF or other genomic feature that is reprogrammable.

### Reprogramming of CpG methylation status between ESCs and adult tissue occurs only at a small fraction of CpGs at binding sites for specific TFs

It has been shown that tissue-specific developmental and adult enhancers have highly methylated CpGs in epiblast but low methylation levels in adult intestinal tissue [50], suggesting that DNA methylation at these sites is reprogrammed. In order to determine the degree to which reprogramming between embryonic and adult somatic tissue occurs, we re-analyzed the data of Jadhav et al. [50] in the context of individual distal CpGs. We chose to look at E7.5 embryos rather than E6.5 epiblast cells since they have globally higher methylation levels and similar methylation patterns, and thus make reprogramming easier to detect. About half of the individual CpGs at ATAC-seq peaks during intestinal development that are highly methylated in E7.5 embryos are lowly methylated in the adult intestine (Additional file 1: Figure S8A). This is consistent with the original published results showing that CpGs at these tissue-specific enhancers are reprogrammed at some point after the epiblast stage [50].

**Fig. 4:**
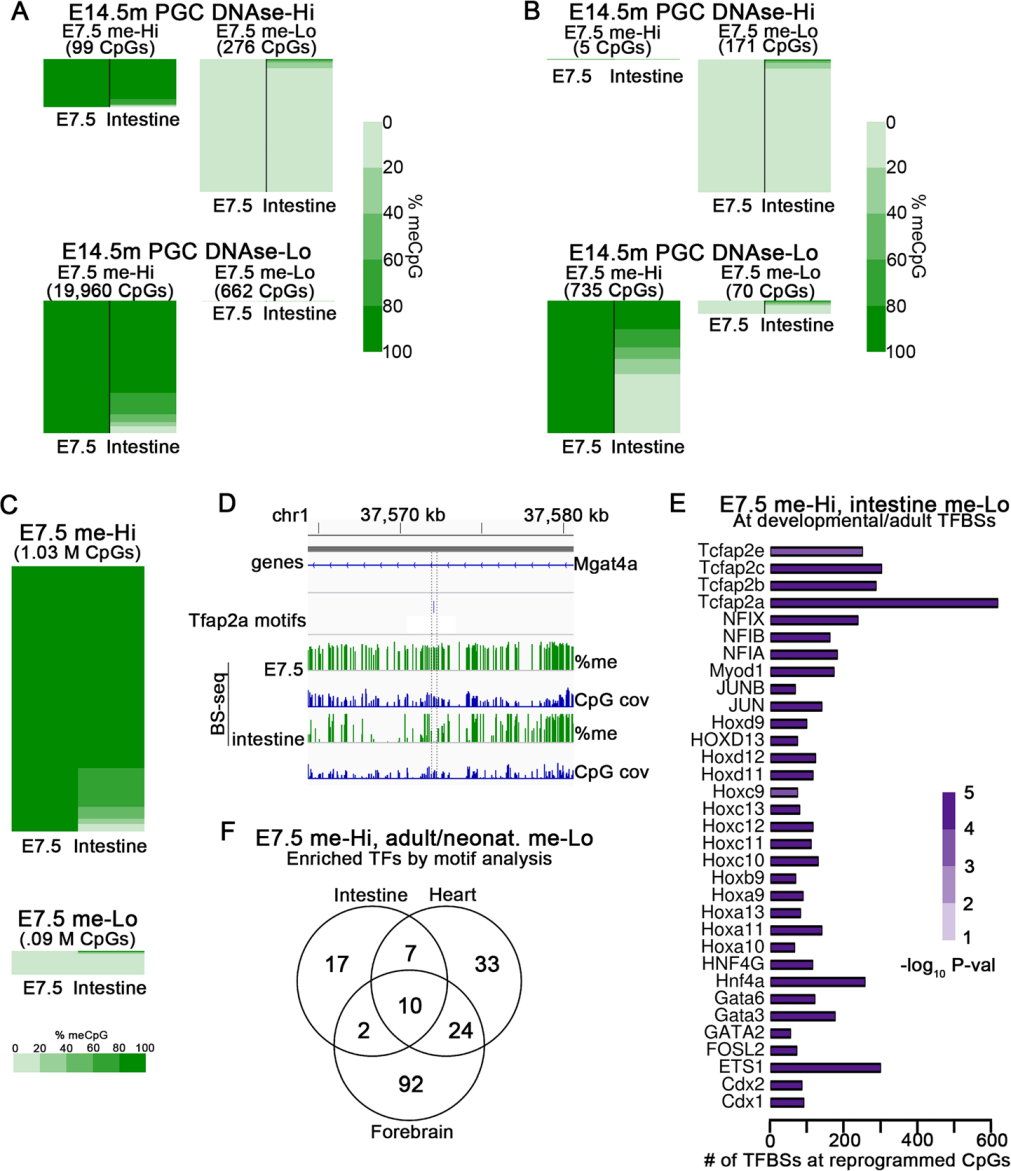
Reprogramming of CpG methylation status between ESCs and adult tissue occurs only at a small fraction of CpGs at binding sites for specific TFs. **a** Heatmap of CpG methylation percentage from BS-seq data in E7.5 embryos and adult intestine. Only CpGs with at least 10 BS-seq reads are displayed. The heatmaps are divided as follows: CpGs at E14.5m PGC DNase-Hi sites with high methylation (> 80%) in E7.5 embryos, sorted by % methylation in intestine (top left); CpGs at E14.5m PGC DNase-Lo sites with very low methylation (< 20%) in E7.5, sorted by methylation in intestine (bottom left); CpGs at E14.5m PGC DNase-Hi sites with very high methylation in E7.5, sorted by methylation in intestine (top right); and CpGs at E14.5m PGC DNase-Lo sites with very low methylation in E7.5, sorted by methylation in intestine (bottom right). The number of CpGs in each category is shown in the matrix to the right. The heatmaps at DNase-Hi sites are scaled separately from those at DNase-Lo sites to allow viewing of each class. The relative heights of the DNase-Hi heatmaps are scaled according to the relative number of CpGs at each, and the DNase-Lo heatmaps are scaled to each other similarly. **b** Heatmap of a subset of the sites shown in **a**, containing only the sites that overlap ATAC-seq peaks during embryonic, fetal, and adult intestine development. **c** Heatmap of all CpGs genome-wide with at least 10 BS-seq reads in both samples that have either high or low methylation in E7.5 embryos. **d** An example of a region with a CpG at a binding sequence for the putative reprogramming TF Tcfap2a that has high methylation in E7.5 and low methylation in the adult intestine. **e** Bar plots showing the number of motifs and the statistical significance of motif enrichment for the indicated TFs. **f** Venn diagram showing the overlap of sites that have high DNA methylation (> 80%) in E7.5 embryos and low methylation (< 20%) in either adult intestine, neonatal heart, or neonatal forebrain

In order to quantify the amount of global CpG methylation reprogramming that occurs during development to adulthood, we looked at the methylation levels in E7.5 embryos and adult intestine at E14.5m PGC DNase-Hi and DNase-Lo TF binding sites (Fig. 4a; Additional file 1: Figure S8B). Of the E14.5m DNase-Lo CpGs with very high methylation levels (> 80%) in E7.5, 70% also have very high methylation levels in the adult intestine. On the other hand, of the E14.5m DNase-Hi CpGs with very low methylation (< 20%) in E7.5 embryos, 91% also have very low methylation in the adult intestine. We performed the same analysis using BS-seq data from the neonatal heart, kidney, and forebrain obtained from ENCODE, and obtained similar results (Additional file 1: Figure S8C-E). These observations suggest that the methylation status of a majority of CpGs at TF binding sites remains consistent between late embryonic and adult tissue, but that some of them are reprogrammed. When restricting the set of E14.5m PGC DNase-Hi and DNase-Lo sites to only those overlapping an ATAC-seq peak present after the epiblast stage during intestine development, at either late embryonic, fetal, or adult stages, a larger amount of reprogramming occurs at DNAse-Lo sites (Fig. 4b), recapitulating the results of Jadhav et al. [50]. This is consistent with the hypothesis that a subset of CpGs bound by TFs after the epiblast stage are highly methylated in the epiblast but then are reprogrammed to become functional during development or in adulthood, but that similar sequences are not reprogrammed when a TF does not bind at some point during development.

In order to determine if reprogramming at TF binding sequences reflects reprogramming of CpGs genome-wide, we examined all CpGs with very high and very low methylation in E7.5 and found that 76% of genome-wide CpGs with very high methylation in E7.5 also have very high methylation in the adult intestine, while 86% of CpGs with very low methylation in E7.5 also have very low methylation in adult intestine (Fig. 4c). These are remarkably similar to the corresponding percentages when considering only TF binding sequences (Fig. 4a), demonstrating that CpG methylation reprogramming occurs only at a small percentage of CpGs genome-wide, or conversely that the methylation status of the majority of CpGs is maintained between embryos and adult somatic tissue. Figure 4d shows an example of a region around a TF binding sequence that is very highly methylated in E7.5 but that has lost its methylation in adult intestine. The fact that the percentage of reprogrammed CpGs increases drastically at TF binding sequences where a TF is known to bind at some point during the course of development from embryos to the adult intestine suggests that this methylation reprogramming may be driven by the binding of reprogramming TFs to methylated DNA to promote their de-methylation.

A number of TFs widely studied in the context of cell differentiation, including Hox, Fos, and Jun family members, are significantly enriched at sites that are reprogrammed between E7.5 embryos and the adult intestine and that bind a TF at some point during the course of development into adult intestinal cells (Fig. 4e). We hypothesize that at least some of these TFs are capable of binding to methylated DNA and subsequently recruiting Tet enzymes to de-methylate nearby CpGs. A similar analysis of neonatal heart, kidney, and forebrain samples from ENCODE reveals sets of TFs with both overlapping and distinct TFs to those of intestine (Fig. 4f; Additional file 1: Figure S8F-H). This suggests that over the course of development, CpGs that are methylated in the embryo become reprogrammed in a tissue-specific manner. Our results suggest that specific reprogramming TFs are likely to be expressed only at specific stages and within specific cell lineages, forming the basis for cell-specific enhancers and other regulatory elements. However, our results suggest that the methylation status of the majority of CpGs in the genome is preserved from embryonic to adult tissues.

### DNAse-Hi sites in E14.5m PGC are more likely to become active enhancers throughout development and in adulthood than DNAse-Lo sites

Given that the DNA methylation status of the majority of CpGs is preserved between E7.5 embryos and adult tissue, we sought to test whether the maintenance of hypomethylation at E14.5m PGC DNAse-Hi sites has a functional impact. To do this, we examined H3K27ac peaks from 22 embryonic and adult tissues produced by the Ren lab at ENCODE [27]. These include data from 15 adult tissues, 5 embryonic tissues, and 2 immortal cell lines. Remarkably, H3K27ac peaks overlap with a significantly larger proportion of E14.5m PGC DNAse-Hi sites than DNAse-Lo sites within all tissues examined (Additional file 1: Figure S9). Around 15% of the DNAse-Hi sites overlap an H3K27ac peak in most tissues, whereas this number for DNAse-Lo sites is only around 3% in most tissues—a roughly 5-fold difference. When all peaks are merged, around 50% of the DNAse-Hi sites overlap an H3K27ac peak vs. only 30% of DNAse-Lo sites (Additional file 1: Figure S9), suggesting that the specific DNAse-Hi and DNAse-Lo sites that become active enhancers are different in each tissue. These results imply that distal sites are more likely to become active enhancers if they are bound by a TF during the PGC and pre-implantation DNA re-methylation phases. They also suggest that sites hypomethylated prior to tissue specification are more likely to bind factors that induce enhancer activation, possibly because more TFs are capable of binding unmethylated DNA than methylated DNA. Although the results of the last section support the idea that TFs can bind to a small number of methylated sites and de-methylate them to create tissue-specific enhancers, the fact that sites are more likely to become active enhancers if they are already hypomethylated in embryos provides support for the idea that hypomethylation of DNA is a cause rather than a consequence of enhancer activation. The E14.5m DNAse-Lo sites were taken from DNAse peaks from various mouse tissues, and thus, the fact that only 30% of DNAse-Lo sites overlap with H3K27ac in the tissues for which there were data suggests that we have not reached a saturation of tissues covering all active enhancers. This would only occur once 100% of DNAse-Lo sites overlap at least one active enhancer in at least one tissue. Thus, it is likely that in the full range of mouse tissues, nearly 100% of E14.5m PGC DNAse-Hi sites will overlap at least one active enhancer in at least one tissue, making it very likely that alterations in TF binding in E14.5m PGCs and ESCs will result in alterations in the phenotype of the adult.

### TF binding site affinity influences the binding patterns of TFs during the DNA re-methylation phases in PGCs and ESCs

We note that in our analyses up to this point, for each of the TFs expressed in PGCs and ESCs, only a fraction of their putative binding sites in the genome are actually bound by a TF. Except for IAPs, CpGs are largely unmethylated in E13.5m PGCs [3] and ICM [4]. In the absence of DNA methylation as an epigenetic determinant—or at least an indicator of an epigenetic determinant—of TF binding in E14.5m PGCs and ESCs, we hypothesize that TFs bind more frequently to high-affinity DNA sequences than to low-affinity ones. To test this, we employed Transcription Factor Affinity Prediction (TRAP), a tool that quantifies TF affinity for a given sequence using biophysical models [51]. The method uses an equation from biophysics to convert the known motif of a TF into the binding energy between a given TF and a given sequence, which TRAP then converts into the expected number of bound TFs [51]. Thus, the TRAP affinity score is interpreted as the expected number of times a given TF will bind to the sequence at a given genomic locus. TRAP has been shown to accurately predict the most likely TF to bind at a given set of regions [52].

**Fig. 5:**
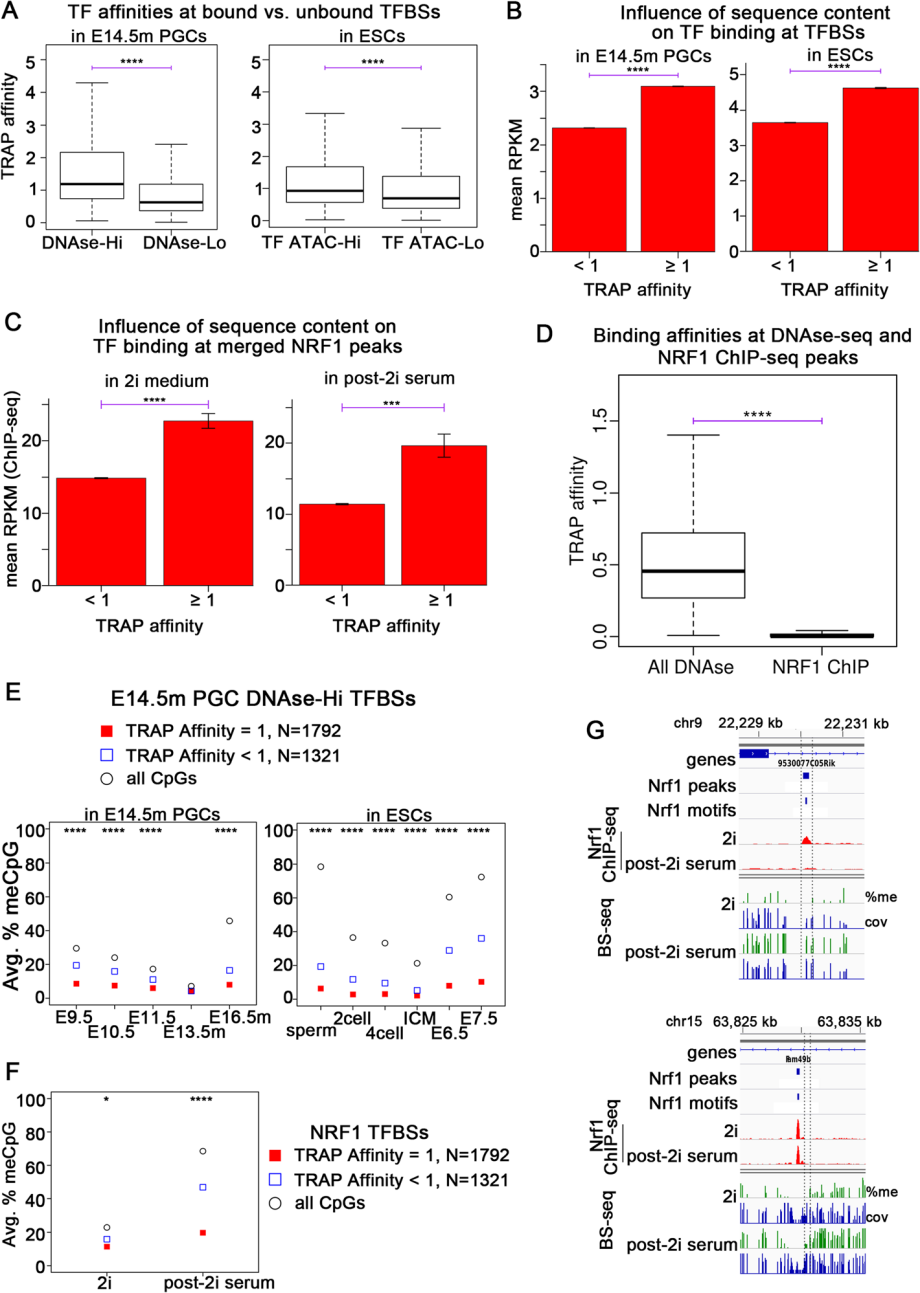
TF binding site affinity influences the binding patterns of TFs during PGC and embryonic development. **a** Boxplots showing the distribution of TRAP affinity scores for TFs at DNase-Hi vs. DNase-Lo sites in E14.5m PGCs and TFs at ATAC-Hi vs. TF ATAC-Lo sites in ESCs. *P* values are by the Wilcoxon rank-sum test. *P* value cutoffs *P < 0.01; **P < 10^−3^; ***P < 10^−5^; ****P < 10^−10^. **b**, **c** Average TF binding signal (DNAse-seq in PGCs and TF ATAC-seq in ESCs) at regions with high and low TRAP affinities in the indicated samples. *P* values are by Student’s *t* test. *P* value cutoffs ***P < 10^−5^; ****P < 10^− 10^. **d** Boxplot comparing TRAP affinity at Nrf1 ChIP-seq peaks to those of DNase-seq peaks. *P* values are by the Wilcoxon rank-sum test. *P* value cutoffs ****P < 10^−10^. **e**, **f** Weighted average DNA methylation levels in the indicated samples at sites with high vs. low TRAP affinity scores for PGC TFs at E14.5m PGC DNase-seq peaks (**e**) and for Nrf1 at Nrf1 ChIP-seq peaks (**f**). *P* values are by Fisher’s exact test. *P* value cutoffs *P < 0.01; **P < 10^−3^; ***P < 10^−5^; ****P < 10^−10^. **g** Example showing the Nrf1 ChIP-seq and DNA methylation levels during and after growth in 2i medium at two Nrf1 peaks, one with a relatively low TRAP affinity (top) and one with a relatively high TRAP affinity (bottom)

We determined a TRAP affinity score for all peaks at DNase-Hi and DNase-Lo sites in E14.5m PGCs. In order to do the same for ESCs, we took all DNAse peaks used to determine DNAse-HI and DNAse-Lo regions, and extracted those that had either high or low TF ATAC-seq signal in ESCs (TF ATAC-Hi and TF ATAC-Lo, respectively). In both E14.5m PGCs and ESCs, the median TRAP affinity was significantly higher in the set of DNAse-Hi sites than the DNAse-Lo sites (Fig. 5a). The difference between TF ATAC-Hi and TF ATAC-Lo sites in ESCs was not as large as for DNAse-Hi vs. DNAse-Lo sites in PGCs. It is possible that this is due to the fact that global CpG methylation levels are lower in E13.5m PGCs than in the ICM, likely resulting in higher methylation levels early on in the re-methylation phase of ESCs than of PGCs, when TFs must bind if not already bound. Methylation-sensitive TFs by definition have a different affinity for DNA at methylated vs. unmethylated CpGs. Since the TRAP affinities are calculated solely based on nucleotide sequence, the actual affinity for TFs would be more different from that predicted by TRAP in ESCs than PGCs due to the globally higher CpG methylation in ESCs than PGCs in the early re-methylation phase. We next combined all TF binding sequences as determined by fimo at all of the multi-tissue set of DNAse-seq peaks extracted from ENCODE, not just those with high and low DNAse-seq signal in E14.5m PGCs, and we divided them according to their TRAP affinity score. The average DNase-seq signal was significantly higher in regions with high TRAP affinity than regions with low TRAP affinity in E14.5m PGCs, and similarly, the average TF ATAC-seq signal was higher in ESCs at regions with high TRAP affinity (Fig. 5b).

It has been shown that binding of Nrf1 in ESCs grown in 2i medium, which results in global CpG de-methylation, can be outcompeted by DNA methyltransferases and become re-methylated after removal from 2i medium [47]. These results could be explained if Nrf1 has a relatively low binding affinity for DNA compared to other TFs. To test this, we computed TRAP affinity scores at all Nrf1 peaks during and after 2i medium exposure. The average ChIP-seq signal was significantly higher at high-affinity peaks in both 2i and standard media (post-2i) (Fig. 5c), consistent with previous TRAP affinity results [52]. Indeed, the median TRAP affinity at Nrf1 peaks was significantly lower than the median TRAP affinity of E14.5m PGC DNase-seq peaks (Fig. 5d). To further test the hypothesis that high-affinity TF binding sites inhibit de novo DNA methylation better than low-affinity sites, we divided the E14.5m PGC DNase-Hi sites into those with high and low TRAP affinity and found that DNA methylation levels were significantly lower at sites with high affinity in both PGCs and ESCs (Fig. 5e). Similarly, we divided Nrf1 ChIP-seq peaks into those with high and low TRAP affinities for Nrf1 and found that after removal from 2i medium, high-affinity Nrf1 binding sites had significantly lower DNA methylation than low-affinity Nrf1 sites (Fig. 5f, g). Taken together, these results suggest that high-affinity binding sites, as determined by TRAP, for TFs expressed in PGC and ESC development, are bound more frequently by their TFs than low-affinity sites and that sites with high affinity will be protected from de novo DNA methylation to a greater extent than low-affinity sites in both PGCs and ESCs.

### IAPs possess a relatively low affinity for embryonic reprogramming TFs and high affinity for non-reprogramming TFs

**Fig. 6:**
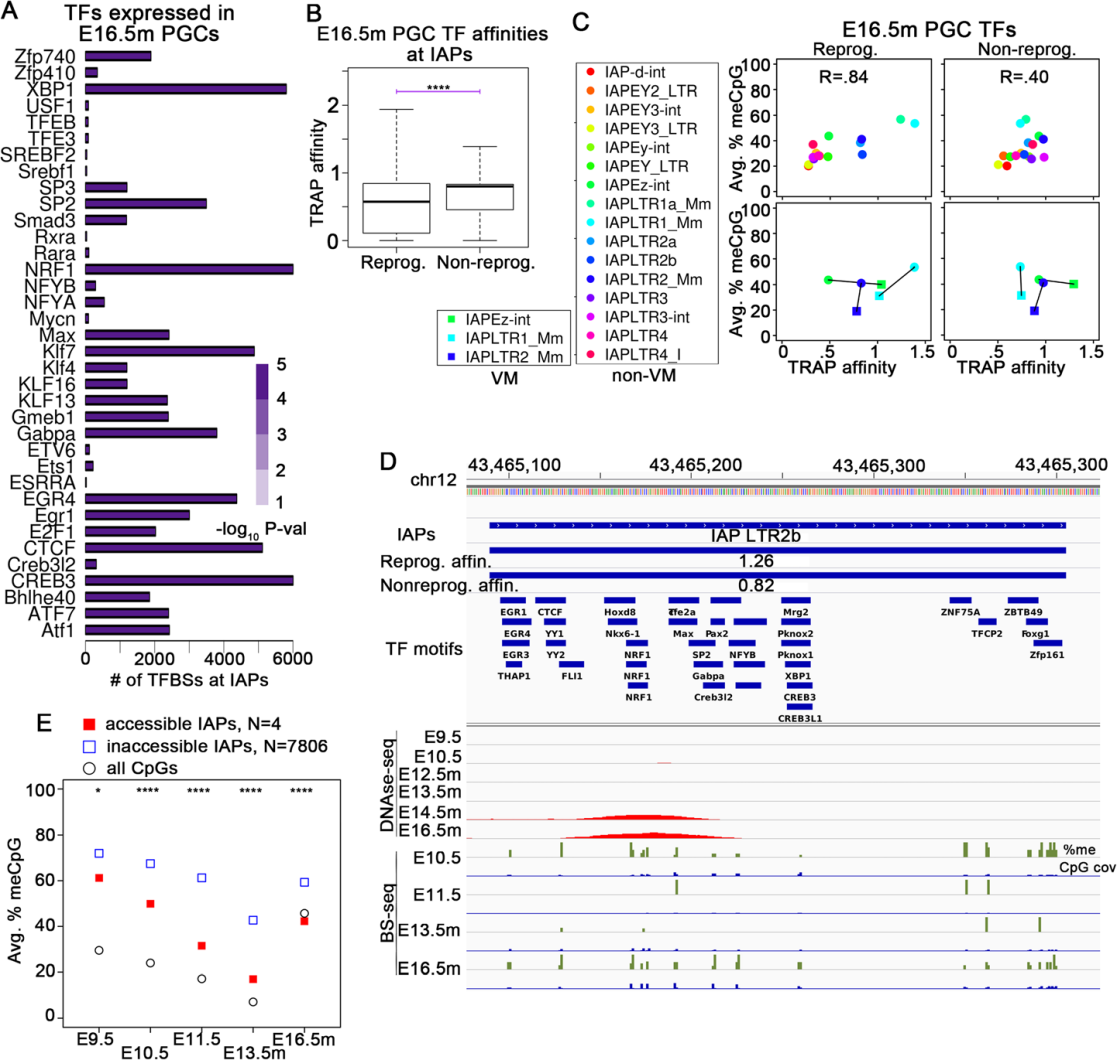
IAPs possess a relatively low affinity for PGC reprogramming TFs and high affinity for non-reprogramming PGC TFs. **a** Bar plots showing the motif enrichment of TFs expressed in E16.5m PGCs at IAP LTR regions. **b** Box plots comparing the TRAP affinity of TFs with evidence of DNA methylation reprogramming in PGCs vs. TFs expressed in PGCs with no evidence of DNA methylation reprogramming capabilities. **c** Scatterplots comparing weighted average CpG methylation in E13.5m PGCs (*y*-axis) to TRAP affinities (*x*-axis) of putative reprogramming TFs expressed in E16.5m PGCs (left column) as well as non-reprogramming TFs expressed in E16.5m PGCs (right column). The top row of scatterplots consists only of non-VM-IAPs while the bottom row of scatterplots compares VM-IAPs to non-VM-IAPs, where a line is drawn between values from VM- and non-VM- IAPs of the same class. **d** Example showing DNase-seq and DNA methylation levels during PGC development at an IAP LTR that has evidence of TF binding. Tracks showing the highest TRAP affinity of the E16.5m PGC putative reprogramming TFs and of the E16.5m PGC non-reprogramming TFs, at the whole IAP, are displayed under the IAP track. **e** Average DNA methylation levels at DNase-seq accessible vs. inaccessible IAPs. The following *P* value cutoffs apply to **b** and **e**: *P < .01; **P < .001; ***P < 10^−5^; ****P < 10^−10^

IAPs are the main class of DNA sequences that remain methylated in E13.5m PGCs [3]. Based on the results described in the previous section, we hypothesized that the DNA binding domain of reprogramming TFs present in PGCs would have a relatively low binding affinity for typical IAP sequences. To test this, we performed a motif enrichment for TFs within annotated IAP long terminal repeats (LTRs) that are expressed in E16.5m PGCs (Fig. 6a). Some TFs associated with reprogramming (Fig. 3g, h) are present in the list of significantly enriched TFs at IAP LTRs (Fig. 6a). We therefore sought to compare the affinity of TFs associated with reprogramming to TFs not associated with reprogramming during DNA re-methylation phases. We hypothesized that if a TF could bind to an IAP at this time, the binding site would be protected from re-methylation just as it is at non-IAP sites. Consistent with this, it has been observed that hypomethylated loci within IAPs are associated with TF binding sites [53]. We limited the analysis to only TFs expressed in E16.5m PGCs. For each IAP LTR, we determined putative reprogramming TFs expressed in E16.5m that have the highest TRAP affinity, and used this information as the reported reprogramming affinity for the corresponding TF binding sites. We similarly determined which non-reprogramming TFs expressed in E16.5m have the highest affinity and used their affinity as the reported affinity for that TF binding sites. We then compared the distribution of these values over all annotated IAP LTRs and found that the median affinity of putative reprogramming TFs was significantly lower than the median affinity for non-reprogramming TFs (Fig. 6b), supporting the hypothesis that IAPs have an inherently low affinity for TFs that can bind to methylated DNA and assist in their de-methylation.

These results suggest that the low affinity for reprogramming TFs may be a contributing factor in helping to ensure that IAPs resist global de-methylation. To further characterize the degree of contribution of IAP DNA sequences to their de-methylation resistance, we plotted the average CpG methylation at E13.5m PGCs vs. the average binding affinity for reprogramming and non-reprogramming TFs expressed in E16.5m PGCs for each class of annotated IAPs in mouse (Fig. 6c). Surprisingly, there is a strong positive correlation (0.84) between CpG methylation levels in E13.5m PGCs and the affinity for putative reprogramming TFs, whereas the correlation with non-reprogramming TF affinities is much weaker (0.40). This suggests that the greater the affinity an IAP has for TFs associated with CpG methylation reprogramming, the greater its resistance to de-methylation during PGC development. This observation suggests that the mechanism of IAP de-methylation resistance is sensitive to the affinity of TFs associated with DNA methylation reprogramming and that one function of this mechanism may be to protect IAPs from binding to reprogramming TFs.

Certain IAPs with variable DNA methylation across individuals but persistent methylation across tissues of an individual are known as variably methylated IAPs (VM-IAPs) [1]. In order to assess the role of TF binding and de-methylation resistance in the formation of VM-IAPs, we compared the average CpG methylation in E13.5m PGCs and the affinity of putative reprogramming and non-reprogramming TFs expressed in E16.5m PGCs at VM vs. non-VM-IAPs of the same class, for the 3 classes of IAPs with at least 25 validated VM-IAPs (Fig. 6c). Interestingly, the TRAP affinity of reprogramming TFs at VM-IAPs appears close to 1 for all 3 classes, and classes of IAPs that have an affinity close to 1 at non-VM-IAPs change very little between their VM and non-VM counterparts, whereas IAPs whose non-VM affinity is smaller than 1 increase to near 1 in their VM counterparts. Furthermore, non-VM-IAPs that have a reprogramming TF affinity larger than 1 decrease to near 1 in their VM-counterparts. Such a relationship does not exist for non-reprogramming TFs (Fig. 6c). These results suggest that VM-IAPs are more likely to form when they have an intermediate affinity for a reprogramming TF. This may be due to the fact that IAPs with a very high affinity for reprogramming TFs are highly resistant to de-methylation in PGCs (Fig. 6b). IAPs with a high level of de-methylation resistance should be able to escape complete de-methylation, whereas IAPs with moderate affinity for reprogramming TFs will have moderate DNA-methylation resistance in PGCs, possibly leading to a stochastic competition between de-methylation and de-methylation resistance in PGCs that results in VM at intermediate values of each. Experimental analyses will be required to directly test this hypothesis. While no obvious relationship between the emergence of VM and the affinity for non-reprogramming TFs emerged in our analysis, their affinities do change between VM and non-VM-IAPs of the same type, suggesting that the final methylation state at IAPs in adults may arise out of a complex interplay between the affinity of reprogramming and non-reprogramming TFs and de-methylation resistance in PGCs and ESCs.

We next looked for IAPs accessible to DNase-seq in PGCs. Although rare, four annotated IAPs were accessible, and these have a relatively high affinity for putative reprogramming TFs (Fig. 6d; Additional file 1: Figure S10A,B). There are likely more than just four TF-bound IAPs genome-wide, but the mappability at IAPs with the DNase-seq data was relatively low since they were obtained by single-end sequencing, while the paired-end BS-seq data is clearly more mappable (Fig. 6d; Additional file 1: Figure S10A,B). In any event, this demonstrates that TFs expressed during PGC development are capable of binding to IAP LTR sequences.

Our results suggest TF binding during the re-methylation phases might result in a trans-generational escape from DNA hypermethylation at the IAP if one or more TFs can bind to high-affinity sites during PGC and ESC development after some event interferes with de-methylation resistance. Indeed, the IAPs accessible to DNase-seq during the re-methylation phase in PGCs have a significantly and substantially lower DNA methylation level throughout PGC development across the entire LTR compared to IAP LTRs that are not accessible to DNase-seq in the re-methylation phase (Fig. 6e). To directly test whether loss of DNA methylation at IAPs results in the binding of TFs, we examined peaks obtained from ChIP-Atlas [54] of Nrf1 in ESCs housed in serum or 2i medium, as well as in Dnmt triple-knockout (TKO) ESCs [47]. A negligible fraction of Nrf1 peaks occurred at IAPs in either the serum or 2i samples, whereas significantly more, ~ 14%, occurred at IAPs in the TKO sample (Additional file 1: Figure S10C). These results suggest that if the affinity of an IAP for a PGC/ESC reprogramming TF can be increased, either by sequence alterations or perhaps by environmentally induced overexpression of a reprogramming TF, it might escape persistently high DNA methylation levels trans-generationally. Interestingly, we found little correlation between the overall mutations at IAPs (see the “Methods” section) and the overall affinity for either reprogramming or non-reprogramming TFs at IAPs (Additional file 1: Figure S10D). This suggests that although a SNP is likely to affect the affinity for TFs in the immediate vicinity, multiple SNPs within an IAP may not affect its overall affinity for TFs. Thus, if a reprogramming TF is able to gain affinity at a specific SNP locus and de-methylate the surrounding region, non-reprogramming TFs would likely be able to bind the surrounding region and possibly protect from re-methylation in successive generations.

## Discussion

Key observations in this study include the fact that the DNA methylation status is faithfully preserved after global de-methylation and re-methylation during the differentiation of the germline and pre-implantation development of the embryo at the majority of CpGs in the mouse genome, and that whether a CpG is methylated or not before and after global de-/re-methylation is highly correlated with the binding of TFs to the genome of PGCs and ESCs. Although it is commonly suggested that trans-generational epigenetic inheritance of DNA methylation can only occur at CpGs that resist de-methylation during PGC and pre-implantation development, results described here suggest that this process occurs even in the absence of de-methylation resistance, because binding of TFs during the re-methylation phase preserves the methylation status between generations.

**Fig. 7:**
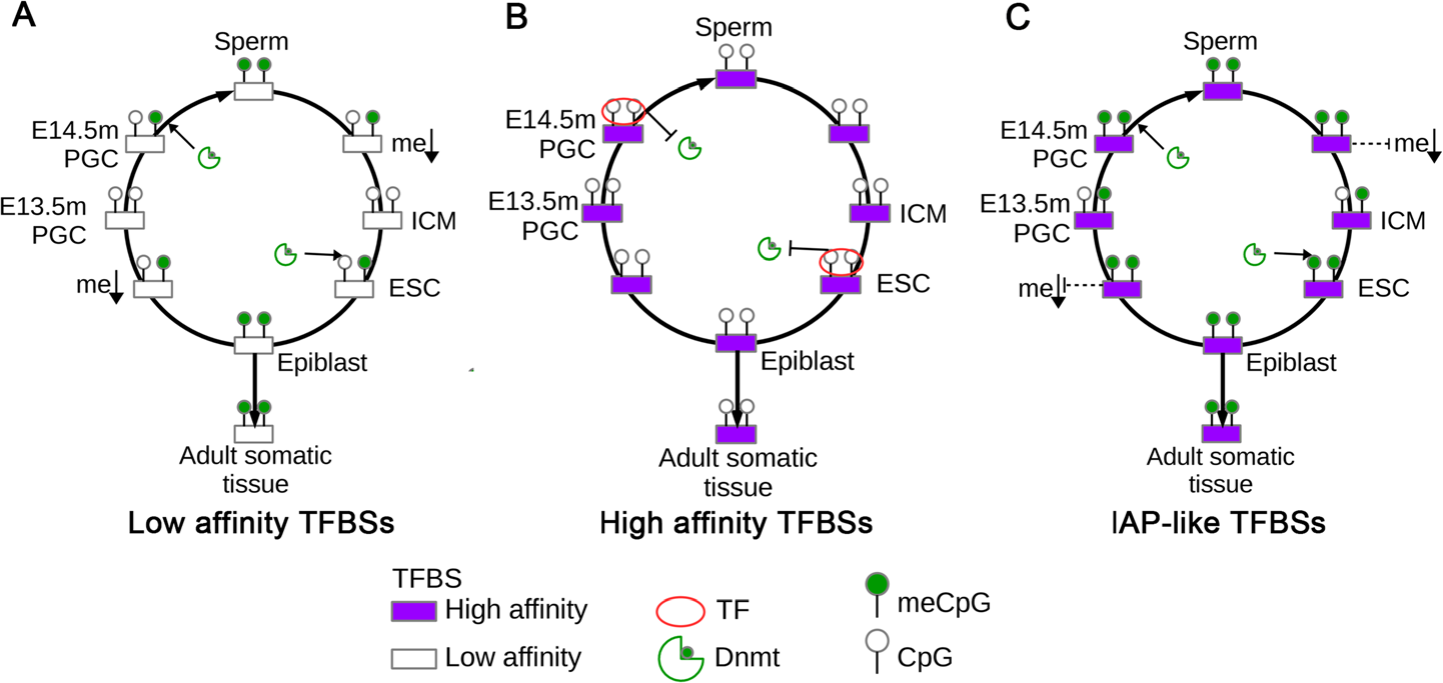
Trans-generational maintenance of CpG methylation status. **a** Methylation dynamics of two CpGs at a locus with low affinity for TFs present in E14.5m PGCs and ESCs. Such sites are hypermethylated in sperm but undergo de-methylation upon fertilization. During the re-methylation phase in ESCs, no TF binds and the site becomes re-methylated. In PGCs, it again becomes de-methylated, only to become re-methylated again starting around E14.5 in male PGCs, in which the site is not bound by a TF. Hypermethylation is maintained in sperm and adult somatic tissues. **b** Methylation dynamics of two CpGs near a high-affinity binding site for at least one TF present in E14.5m PGCs and ESCs. The site is hypomethylated in E14.5m PGCs and is bound by a TF; it remains hypomethylated through sperm maturation. This hypomethylated state is passed to the next generation upon fertilization. In ESCs, this site again binds a TF and subsequently remains hypomethylated. The site is hypomethylated in successive generations in both sperm and adult somatic tissues. **c** Methylation dynamics of a pair of CpGs at an IAP-like DNA element. These sites are capable of resisting global de-methylation, at least in part, upon fertilization and in PGCs. They remain partially methylated in the ICM and E13.5m PGCs and are hypermethylated at all other stages of development. These sites tend to have a high affinity for TFs in E14.5m PGCs and ESCs, but rarely become bound by them, possibly due the high DNA methylation levels. Thus, they remain persistently hypermethylated in sperm and adult somatic tissues

Non-IAP-like sites with low affinity for TFs remain unbound during the PGC and ESC re-methylation phase and are not protected from de novo methylation. The methylation state of such sites is persistently high across generations in epiblasts, adult sperm, and adult somatic tissue, despite two phases of global de-methylation followed by re-methylation per generation (Fig. 7a). Only a small percentage of such sites become de-methylated in certain adult tissues due to reprogramming. At non-IAP-like sites, CpGs near high-affinity TF binding sites bind their cognate TFs early on in the re-methylation phase and are subsequently protected from de novo methylation (Fig. 7b). At IAP-like sites, CpGs remain persistently hypermethylated throughout development and across generations in sperm and adult somatic tissue despite having a relatively high affinity for non-reprogramming TFs (Fig. 7c). IAPs are the only class of DNA sequences shown to escape de-methylation followed by re-methylation during PGC development in mice, although de-methylation resistance has been also observed at a small number of non-IAP CpGs [3]. Consistent with persistent hypermethylation at IAPs, TFs rarely bind to IAPs—even at high-affinity binding sites—in the germline (Fig. 7c).

Given that IAPs typically have a high affinity for non-reprogramming TFs, our model predicts that an environmental stimulus capable of interfering with the ability of an IAP to resist de-methylation during PGC or pre-implantation development would enable TFs to bind and protect it from methylation during the subsequent re-methylation phase. This would result in a trans-generational switch from persistently hypermethylation to persistent hypomethylation because once the region becomes hypomethylated, TFs will be able to bind in every re-methylation phase and protect it from being methylated. This is equivalent to changing from behaving as in Fig. 7c to behaving as in Fig. 7b. An obvious way in which this could happen is through a mutation that ablates the de-methylation resistance but still leaves a high affinity for a TF, which is likely to occur when mutations happen at IAPs. Alternatively, if a reprogramming TF can bind to the IAP and de-methylate it, a similar result would occur. IAPs tend to have a low affinity for such reprogramming TFs, but an environmental stimulus causing ectopic overexpression of such a TF may allow it to bind. Esrrb is expressed in PGCs and has been shown to bind methylated DNA and promote its de-methylation [46]. Our study has identified Esrra as a TF potentially capable of reprogramming the inter-generational methylation pattern of CpGs. Ectopic expression of Esrra has been observed in response to exposure to the endocrine disruptor BPA [55], and exposure to BPA during embryonic development has been shown to induce the viable yellow phenotype in *Agouti viable yellow* mice via loss of DNA methylation [55]. The Agouti viable yellow phenotype is known to be induced by de-methylation of the IAP upstream of the *Agouti* gene. Esrrb was also shown to displace nucleosomes and induce a more permissive chromatin state when binding to methylated DNA [46]. This is important because, although it has been shown that depletion of DNA methylation results in expression of IAPs and other transposable elements [56, 57], the aberrant expression has been shown to be subsequently reduced by the accumulation of repressive histone modifications [58]. We must note the caveat that this subsequent reduction in expression occurred in 2i media, in which IAPLTR1 and IAPLTR2 elements remain methylated. However, even in the event that such a mechanism occurs in IAPs, the binding of a reprogramming TF like Esrrb would likely displace nucleosomes with such repressive marks and enable TFs to bind nearby and possibly sustain the local nucleosome displacement. Esrra and Esrrb therefore represent promising candidates for TFs capable of inducing trans-generational changes in phenotype when ectopically overexpressed during PGC or pre-implantation development.

piRNAs are known to increase methylation levels at IAPs [59]. However, it is not clear whether this mechanism is active during global de-methylation of PGCs and pre-implantation embryos [60]. IAPs are partially de-methylated during PGC and pre-implantation development, suggesting that the mechanism of IAP DNA methylation maintenance competes with global de-methylation rather than completely ablating it. Alterations in miRNAs and tRNA fragments have also been shown to induce inter- or trans-generational changes in phenotype. Both have the potential to regulate retroelements such as IAPs by affecting the translation of proteins involved in their regulation, and alteration of these RNAs has been linked to heritable epigenetic changes [10, 11, 13]. This suggests that one possible mechanism by which ncRNAs can induce trans-generational epigenetic effects is by interfering with the ability of IAPs to resist de-methylation during PGC and pre-implantation development. An increase in miRNAs could inhibit the translation of proteins needed to maintain methylation at IAPs, while a loss of tRNAs could have the same effect. Indeed, alterations in piRNAs have been observed in studies of trans-generational inheritance of stress-induced epiphenotypes [10], which, as discussed, are thought to regulate DNA methylation at IAPs and other transposable elements. Thus, the alterations in miRNAs are coincident with likely alterations in de-methylation resistance. In addition, sperm tRNAs are involved in downregulating expression of the MERVL transposable element in pre-implantation embryos in studies showing the involvement of tRNA fragments in low protein diet-induced trans-generational epiphenotypes [13]. These studies also observed that the levels of several piRNAs were altered in sperm but did not explore this observation further. Nonetheless, these studies establish a link between ncRNAs and regulation of transposable element de-methylation resistance in inter- and trans-generational epigenetic inheritance [13].

ncRNA-mediated inhibition of transcription or translation might also directly affect the levels of TFs during the DNA re-methylation. Although this would have widespread effects on transcription and chromatin, our model predicts that only changes in DNA methylation at IAPs and downstream effects will be maintained trans-generationally. Although ncRNAs have been shown to be able to induce trans-generational changes in phenotype, to date, there is no evidence that they are responsible for maintaining the altered phenotype trans-generationally. On the contrary, as we have already discussed, there is evidence that ncRNAs are not involved in trans-generational maintenance of the phenotype they initially induce [10]. Thus, it is likely that initial changes in transcription/translation of TFs will not be maintained beyond the F_2_ generation, whereas a loss of methylation at IAPs due to the initial change in TF levels would likely be maintained because IAPs have a high affinity for multiple TFs that are present in abundance in E14.5m PGCs and ESCs. Thus, even when the TFs altered by ncRNAs are restored to normal levels, once the IAP is hypomethylated, it would be able to bind other constitutive TFs and remain protected from re-methylation.

Our model predicts that trans-generational changes in phenotype can occur via ectopic inhibition of de-methylation resistance followed by maintenance of hypomethylation at IAP-like sites (Additional file 1: Figure S11A,B), whereas inter-generational epigenetic inheritance could occur via ectopic alterations to any number of epigenetic carriers, including ncRNAs or histone modifications that result in downregulation of Dnmt or TF activity during the DNA re-methylation phase at non-IAP loci (Additional file 1: Figure S11C-F). What makes IAPs unique from other genomic loci is that they are typically hypermethylated but have a high affinity for TFs that cannot normally bind, likely due to DNA hypermethylation. However, once ectopically hypomethylated, they can maintain the hypermethylated site due to their high affinity for re-programming TFs, which will likely protect them from subsequent re-methylation. On the other hand, an ectopic change in a ncRNA or non-IAP CpGs would simply be restored back to their original state in the next generation because the input that induced the change is no longer present, and there is no known mechanism for their maintenance trans-generationally. In a study of the inter-generational effects of paternal diet, Shea et al. found that variations in sperm DNA methylation do not play a major role [61]. This is consistent with our model since it predicts two mechanisms by which changes in DNA methylation of F_1_ adult somatic tissue can occur without changes in methylation in F_1_ sperm. Changes in a ncRNA or other factor in the F_0_ sperm would result in an inter-generational change in phenotype if it could persist through the ICM and inhibit re-methylation or the abundance of a TF. Alternatively, an altered ncRNA or other factor could simply induce phenotypic changes independent of DNA methylation in the pre-implantation embryo that could have effects into adulthood. Either scenario is consistent with our model. Only trans-generational inheritance strictly requires DNA methylation as a mediator, according to our model, and consistent with this, the best studied cases of trans-generational epigenetic inheritance are known to be mediated by DNA methylation [5–8].

There is evidence that specific histone modifications such as H3K4me3 can act as mediators that protect CpGs from de novo methylation [18]. Here, we observe that TF binding sites during re-methylation phases are enriched for Mll2, Ash2l, and Tet1 in ESCs and are flanked by H3K4me3 in PGCs and ESCs. It has been shown that binding of pioneer TFs can direct nucleosome positioning [62]. Thus, the evidence suggests that one way in which TFs protect from methylation may by the placement of H3K4me3 and the recruitment of a range of co-factors that promote a hypomethylated state on DNA. Importantly, there is solid evidence that TFs play a causal role in protecting from de novo DNA methylation [41, 63], suggesting multiple, possibly redundant, modes of hypomethylation maintenance. Taken together, our results demonstrate that TFs are capable of maintaining a hypomethylated state of CpGs near their binding sites trans-generationally.

## Conclusion

The results of this study support models explaining the occurrence of inter- and trans-generational epigenetic inheritance via TF binding during global DNA re-methylation of PGCs and pre-implantation embryos. Our results can explain the preservation of CpG methylation status despite two rounds of global de-methylation and re-methylation per generation. These models predict that if re-methylation or TF binding were inhibited at non IAP-like genomic loci, this would only result in inter- rather than trans-generational inheritance because the ectopic change would not persist to the next re-methylation phase. On the other hand, our model predicts that inhibition of de-methylation resistance at an IAP would result in a trans-generational shift from hypermethylated to hypomethylated DNA in the region that becomes initially de-methylated. The model therefore provides a framework for the design of experiments intended to elucidate specific mechanisms of inter- and trans-generational epigenetic inheritance.

## Methods

### DNase-seq and ChIP-seq analysis

Raw fastq files were downloaded from the sources cited in the text and trimmed using Trimmomatic-0.38 [64] with the parameters “1:0:2 TRAILING:20 MINLEN:20.” Trimmed reads were mapped with bowtie [65] with the parameters -m 1 --mapq 254. Duplicate reads were then removed using MarkDuplicates from picard-tools version 2.1.0 (https://broadinstitute.github.io/picard/). For displaying in IGV, macs2 [16] predictd was used to determine the average fragment length, and mapped reads were shifted by half the average predicted fragment length towards the fragment center. The genome-wide, normalized coverage was then determined using bedtools [66] genomecov on the deduplicated, shifted reads, scaled by the total number of processed reads per million in each sample.

### ATAC-seq analysis

Raw fastq files were downloaded from the sources cited in the text and trimmed using pyadapter_trim.py (https://github.com/kundajelab/training_camp/blob/master/src/pyadapter_trim.py). To adjust the fragment size for transposase insertions, we aligned all reads as + strands offset by + 4 bp and − strands offset by 5 bp [67]. Trimmed and offset reads were aligned to the mm9 reference genome using bowtie2 [68] with the parameter -X 2000. Only paired-end reads with fragment length between 50 and 115 bp, corresponding to TF binding sites [67], referred to in this paper as TF ATAC, were kept. For viewing in IGV, a bed file of processed TF fragments was created, and the genome-wide, normalized coverage was then determined using bedtools genomecov, scaled by the total number of processed TF reads per million in each sample.

### BS-seq analysis

For the embryonic data [4], processed files containing read count information of methylated and unmethylated CpGs were downloaded and converted from mm10 to mm9 using liftover [69]. For all other BS-seq data, raw fastq files were downloaded and trimmed as described for the DNase-seq and ChIP-seq data. Trimmed reads were then aligned to the mouse mm9 reference genome using bismark [70] v0.19.0, deduplicated with deduplicate_bismark, and then CpG methylation was extracted using bismark_methylation_extractor. For each CpG, the number of meCpG and CpG reads for all replicates of a given sample was combined to obtain a single average methylation value per sample per CpG. For heatmaps, the R function pheatmap was used, and only CpGs with > 10 BS-seq reads were used. For plots of average methylation, the average was calculated using all CpGs, with each CpG being weighted by the total number of BS-seq reads at that CpG. This is equivalent to simply pooling all BS-seq reads at the CpGs being considered at taking the overall average.

### RNA-seq analysis

Raw fastq files were downloaded from the sources cited in the text, and reads were trimmed as described in the “DNase-seq and ChIP-seq analysis” section. Reads were then aligned to the mm9 reference genome using tophat2 [71] with the parameters --no-mixed --no-discordant, and non-uniquely mapped reads were discarded. FPKM values for annotated genes were calculated using cuffdiff.

### Estimation of the percentage of CpGs with a preserved methylation status prior to and after global de-methylation and re-methylation of PGCs and pre-implantation embryos

Only 9% of CpGs in sperm, or 0.15 million CpGs, had intermediate methylation levels (between 20 and 80%), and we conservatively assume that none of those will be preserved before and after PGC and pre-implantation reprogramming. Of the 1.38 million CpGs with > 80% methylation in sperm, 91% had > 50% methylation in epiblast, but since the epiblast is still undergoing de novo methylation, we assume that anything over 50% in epiblast should eventually become over 80% methylated in sperm and so we consider that these are CpGs whose methylation status is preserved before and after PGC and pre-implantation reprogramming. The remaining 0.18 million CpGs in sperm have < 20% methylation, and of these, 46% also have < 20% methylation in epiblast, so we consider that 46% are preserved. Based on these assumptions, the percentage of preserved CpGs is given by the weighted average of the percentages of preserved CpGs out of those that are very high in sperm, intermediate in sperm, and very low in sperm, as described above. Explicitly, the calculation is as follows: (1.38 × 0.91 + 0.18 × 46 + 0.15 × 0)/(1.38 + 0.18 + 0.15).

### Estimation of TF binding affinities based on DNA sequence

Transcription Factor Affinity Prediction [51] scores were calculated using TEPIC [72] at PGC DNase-seq peaks downloaded from NCBI GEO [23] as well as at annotated IAP LTRs. An affinity score was calculated for all TFs in the “Merged_JASPAR_HOCOMOCO_KELLIS_Mus_musculus.PSEM” file provided with the TEPIC software. For each region where TRAP affinities were calculated, the maximum TRAP affinity of the TFs under consideration was chosen as the affinity score used for that region in subsequent analyses, since in PGCs, at any given TFBS for an embryonic TF, the TF with the highest binding affinity is likely to be the one that binds to that region.

### Quantification of mutations at IAPs

At each class of IAP, the Levenshtein edit distance was used to compare IAP LTR sequences in the mm9 reference to the consensus LTR sequence obtained from Dfam [73]. Edit distances were calculated by the R function stringdist(). Because the consensus sequences contained more repeats and so were much larger in size than the annotated IAP sequences, the difference in size between the consensus and the specific sequence being compared was subtracted from the Levenshtein distance to obtain the final edit distance value used. These values were log transformed for plotting and correlation analysis.

## Supplementary information

**Additional file 1: Figure S1.** TF binding, mRNA expression, and DNA methylation in PGCs (Related to Figure 1). **Figure S2**. Average DNAse-seq footprint profiles in E14.5m PGCs at E14.5m PGC DNAse-Hi sites containing binding sequences for the indicated TFs (Ralated to Fig. 1). **Figure S3.** Additional Data on E14.5m PGC DNAse-Hi sites (Related to Fig. 1). **Figure S4.** TF binding and DNA methylation of sperm and pre-implantation embryos at E14.5m PGC DNAse-Hi and -Lo sites (Related to Fig. 2). **Figure S5**. Average ATAC-seq footprint profiles in ESCs at E14.5m PGC DNAse-Hi sites containing binding sequences for the indicated TFs (Related to Fig. 2). **Figure S6.** Co-factors at E14.5m PGC DNAse-Hi sites (Related to Fig. 2). ** Figure S7.** DNA methylation, co-factor binding, and mRNA expression related to E9.5-trace, E14.5m-Hi and E9.5-Hi, E14.5m-trace sites (Related to Fig. 3). **Figure S8.** DNA methylation maintenance from E7.5 embryos to neonatal and adult tissues (Related to Fig. 4). **Figure S9.** Overlap of E14.5m PGC DNAse-Hi and DNAse-Lo sites with H3K27ac peaks from the indicated tissue (Related to Fig. 4). **Figure S10.** TF binding and sequence content at IAPs (Related to Fig. 6). **Figure S11.** Predicted mechanisms of inter- and trans-generational epigenetic inheritance (Related to Fig. 7).

**Additional file 2: Table S1.** TFs whose motifs are enriched at E14.5m PGC DNAse-Hi sites that are expressed in E16.5m PGCs based on RNA-seq.

**Additional file 3.** Genes associated with the binding of reprogramming TFs. Left column: genes with FPKM > 10 in E11.5 PGCs within 10 kb of an E9.5-Hi, E14.5m-trace site. Right column: genes with FPKM > 10 in E16.5m PGCs within 10 kb of an E9.5-trace, E14.5m-Hi site.

**Additional file 4.** Review history.

## Data Availability

All datasets analyzed in the present study are available in either the NCBI GEO, ENCODE, or European Nucleotide Archive repositories at the following locations: Epiblast and PGC DNase-seq, GSE109770 [23]; PGC BS-seq, PRJEB3376 [3]; Preimplantation DNase-seq, GSE76642 [24]; Preimplantation and Sperm BS-seq, GSE56697 [4]; RS ATAC-seq, GSE102954 [26]; Sperm ATAC-seq, GSE116857 [20]; ICM ATAC-seq, GSE66390 [74]; ESC ATAC-seq, GSE67299 [25]; Adult intestine ATAC-seq and BS-seq, GSE111024 [50]; Neonatal forebrain BS-seq, GSE82356 [27]; Neonatal heart BS-seq, GSE82658 [27]; Neonatal kidney BS-seq, GSE82451 [27]; NRF1 ChIP-seq and BS-seq in 2i and standard serum, GSE67867 [47]; Mouse H3K27ac peaks, GSE31039 [27]; Mouse DNase-seq peaks, http://hgdownload.soe.ucsc.edu/goldenPath/mm9/encodeDCC/wgEncodePsuDnase/ [27]; and Mouse DNase-seq peaks, http://hgdownload.soe.ucsc.edu/goldenPath/mm9/encodeDCC/wgEncodeUwDnase/ [27]. All custom scripts used to process and analyze the data and to obtain the results described in this paper have been deposited in the GitHub repository (https://github.com/ikremsky/Scripts-for-Kremsky-Corces-2020-Genome-Biology-paper) [74].
