# Supplementary material for: Protection from DNA re-methylation by transcription factors in primordial germ cells and pre-implantation embryos can explain trans-generational epigenetic inheritance
Source: Genome Biol. 2020 May 18;21:118. doi: 10.1186/s13059-020-02036-w (PMC7236515; doi:10.1186/s13059-020-02036-w)
Supplement: Supplementary file 1 — Additional file 1: Figure S1. TF binding, mRNA expression, and DNA methylation in PGCs (Related to Figure 1). Figure S2. Average DNAse-seq footprint profiles in E14.5m PGCs at E14.5m PGC DNAse-Hi sites containing binding sequences for the indicated TFs (Ralated to Fig. 1). Figure S3. Additional Data on E14.5m PGC DNAse-Hi sites (Related to Fig. 1). Figure S4. TF binding and DNA methylation of sperm and pre-implantation embryos at E14.5m PGC DNAse-Hi and -Lo sites (Related to Fig. 2). Figure S5. Average ATAC-seq footprint profiles in ESCs at E14.5m PGC DNAse-Hi sites containing binding sequences for the indicated TFs (Related to Fig. 2). Figure S6. Co-factors at E14.5m PGC DNAse-Hi sites (Related to Fig. 2). Figure S7. DNA methylation, co-factor binding, and mRNA expression related to E9.5-trace, E14.5m-Hi and E9.5-Hi, E14.5m-trace sites (Related to Fig. 3). Figure S8. DNA methylation maintenance from E7.5 embryos to neonatal and adult tissues (Related to Fig. 4). Figure S9. Overlap of E14.5m PGC DNAse-Hi and DNAse-Lo sites with H3K27ac peaks from the indicated tissue (Related to Fig. 4). Figure S10. TF binding and sequence content at IAPs (Related to Fig. 6). Figure S11. Predicted mechanisms of inter- and trans-generational epigenetic inheritance (Related to Fig. 7). [file 13059_2020_2036_MOESM1_ESM.pdf]

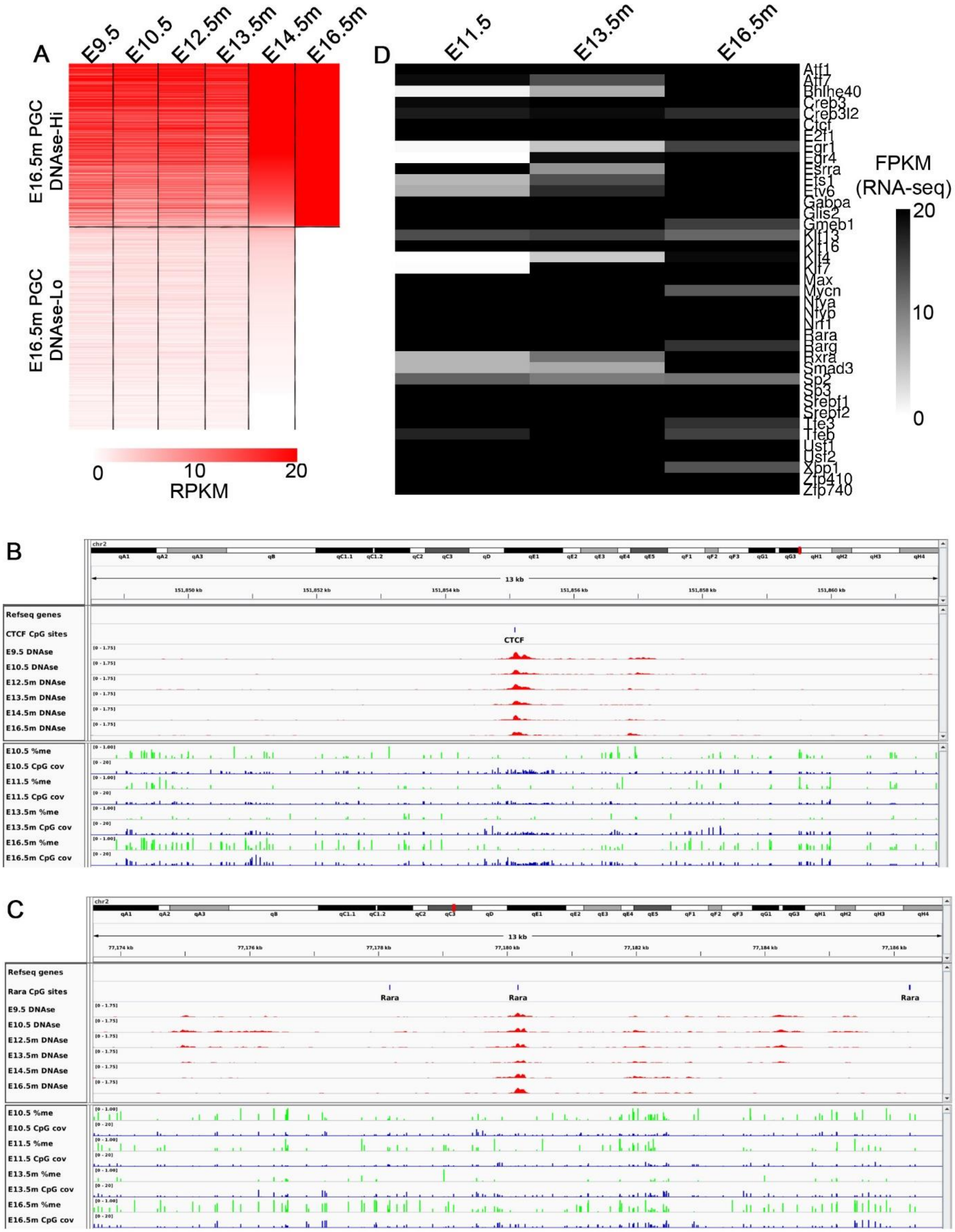

A
E9.5
E10.5
E12.5m
E13.5m
E14.5m
E16.5m
E16.5m PGC DNAse-Hi
E16.5m PGC DNAse-Lo
0
10
20
RPKM
D
E11.5
E13.5m
E16.5m
Atf1
Atf7
Bhlhe40
Creb3
Creb3l2
Ctcf
E2f1
Egr1
Egr4
Esrra
Ets1
Etv6
Gabpa
Glis2
Gmeb1
Klf13
Klf16
Klf4
Klf7
Max
Mycn
Nfya
Nfyb
Nrf1
Rara
Rarg
Rxra
Smad3
Sp2
Sp3
Srebf1
Srebf2
Tfe3
Tfeb
Usf1
Usf2
Xbp1
Zfp410
Zfp740
FPKM (RNA-seq)
20
10
0
B
chr2
13 kb
Refseq genes
CTCF CpG sites
CTCF
E9.5 DNAse
E10.5 DNAse
E12.5m DNAse
E13.5m DNAse
E14.5m DNAse
E16.5m DNAse
E10.5 %me
E10.5 CpG cov
E11.5 %me
E11.5 CpG cov
E13.5m %me
E13.5m CpG cov
E16.5m %me
E16.5m CpG cov
C
chr2
13 kb
Refseq genes
Rara CpG sites
Rara
Rara
Rara
E9.5 DNAse
E10.5 DNAse
E12.5m DNAse
E13.5m DNAse
E14.5m DNAse
E16.5m DNAse
E10.5 %me
E10.5 CpG cov
E11.5 %me
E11.5 CpG cov
E13.5m %me
E13.5m CpG cov
E16.5m %me
E16.5m CpG cov

Figure S1. TF binding, mRNA expression, and DNA methylation in PGCs. **A** Heatmap of RPKM values within just binding sequences called by fimo, separated into those that have high DNase-signal in E16.5m PGCs (DNase-Hi, RPKM > 20) and those that have RPKM=0 (DNase-Lo). Rows are ordered by decreasing signal in of E14.5m. All stages shown are during PGC development. **B,C** Example region with DNase accessibility and low local DNA methylation throughout PGC development. CpG cov indicates the BS-seq read coverage. TF binding sequences called by fimo that overlap the peak summit in E14.5m are shown. **D** RNA-seq levels in the indicated stages of PGC development for TFs that are expressed in E16.5m PGCs and were significantly enriched in a motif analysis at E14.5m PGC DNAse-Hi sites. (Related to Figure 1).

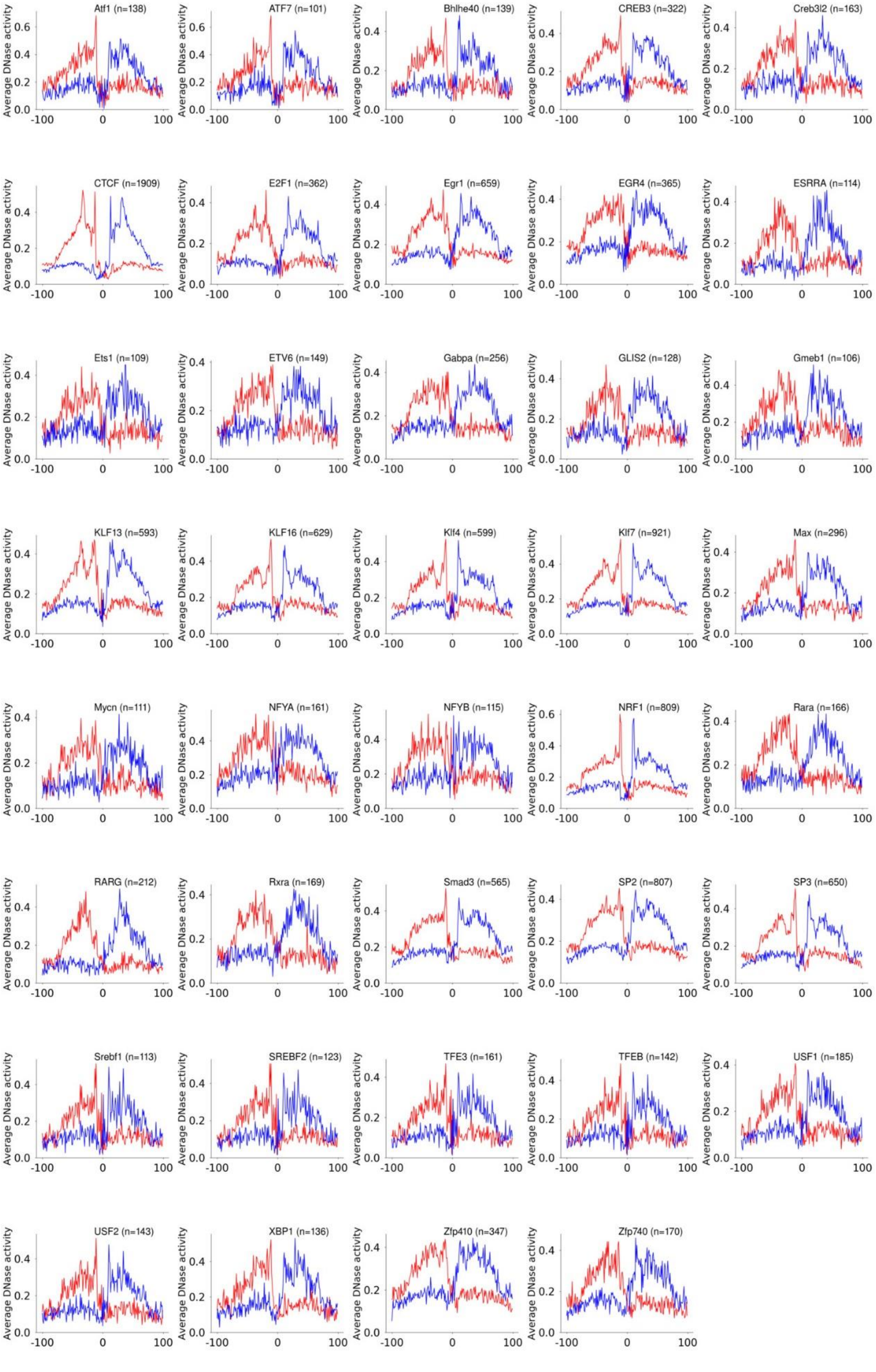
Atf1 (n=138)
ATF7 (n=101)
Bhlhe40 (n=139)
CREB3 (n=322)
Creb3l2 (n=163)
CTCF (n=1909)
E2F1 (n=362)
Egr1 (n=659)
EGR4 (n=365)
ESRRA (n=114)
Ets1 (n=109)
ETV6 (n=149)
Gabpa (n=256)
GLIS2 (n=128)
Gmeb1 (n=106)
KLF13 (n=593)
KLF16 (n=629)
Klf4 (n=599)
Klf7 (n=921)
Max (n=296)
Mycn (n=111)
NFYA (n=161)
NFYB (n=115)
NRF1 (n=809)
Rara (n=166)
RARG (n=212)
Rxra (n=169)
Smad3 (n=565)
SP2 (n=807)
SP3 (n=650)
Srebf1 (n=113)
SREBF2 (n=123)
TFE3 (n=161)
TFEB (n=142)
USF1 (n=185)
USF2 (n=143)
XBP1 (n=136)
Zfp410 (n=347)
Zfp740 (n=170)
Average DNase activity

Figure S2. Average DNAse-seq footprint profiles in E14.5m PGCs at E14.5m PGC DNAse-Hi sites containing binding sequences for the indicated TFs (Related to Figure 1).

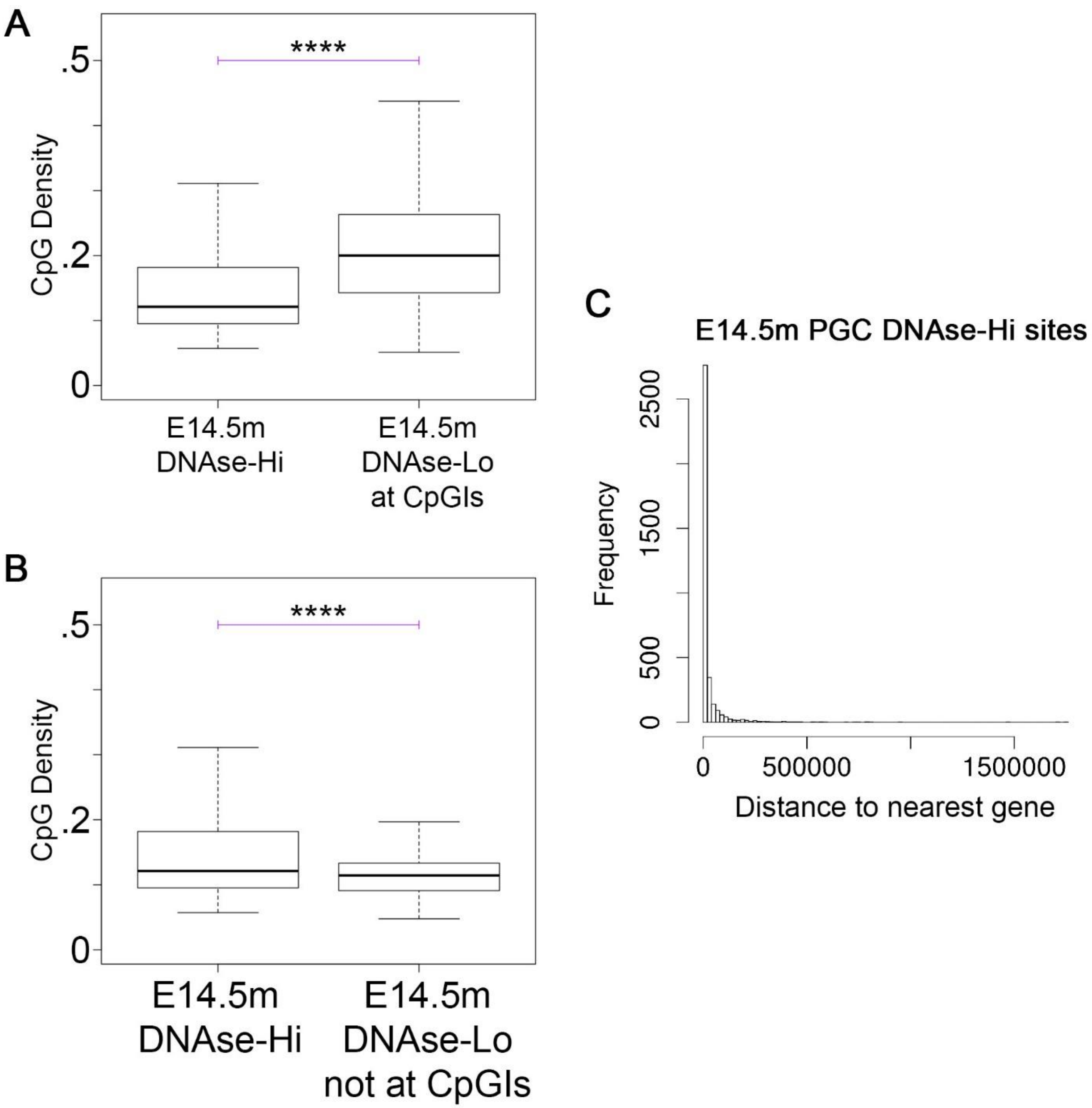

Figure S3. Additional Data on E14.5m PGC DNAse-Hi Sites. A Boxplots comparing the distribution of CpG densities at E14.5m PGC DNAse-Hi sites to the distribution at DNAse-Lo sites within a CpG island (CPGI). B Boxplots comparing the distribution of CpG densities at E14.5m PGC DNAse-Hi sites to the distribution at DNAse-Lo sites not at a CpGI. P-vals in A and B were calculated by the Wilcoxon Test and are indicated as follows: *P<.01; **P<.001; ***P<10^{-5}; ****P<10^{-10}. C Histogram of distances (in nucleotides) from E14.5m PGC DNAse-Hi sites to the nearest gene. (Related to Figure 1).

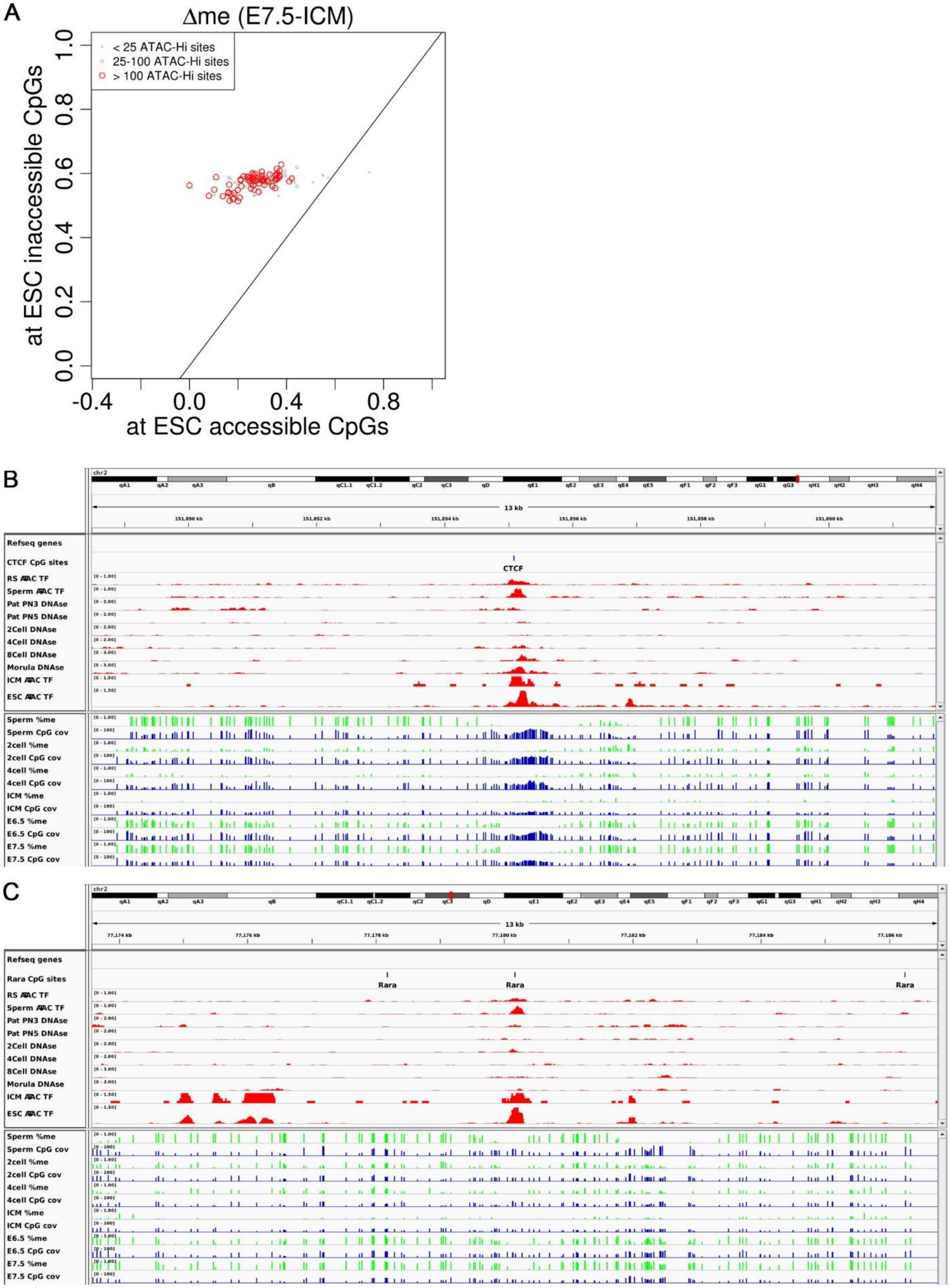
A
Δme (E7.5-ICM)
< 25 ATAC-Hi sites
25-100 ATAC-Hi sites
> 100 ATAC-Hi sites
at ESC inaccessible CpGs
at ESC accessible CpGs
B
chr2
13 kb
CTCF CpG sites
CTCF
C
chr2
13 kb
Rara CpG sites
Rara
Refseq genes
RS ATAC TF
Sperm ATAC TF
Pat PN3 DNAse
Pat PN5 DNAse
2Cell DNAse
4Cell DNAse
8Cell DNAse
Morula DNAse
ICM ATAC TF
ESC ATAC TF
Sperm %me
Sperm CpG cov
2cell %me
2cell CpG cov
4cell %me
4cell CpG cov
ICM %me
ICM CpG cov
E6.5 %me
E6.5 CpG cov
E7.5 %me
E7.5 CpG cov

Figure S4. TF binding and DNA methylation of sperm and pre-implantation embryos at E14.5m PGC DNAse-Hi and -Lo sites. **A** Scatterplot of changes in DNA methylation between E14.5m and E16.5m PGCs. Each point represents the average methylation change (fraction meCpG in E16.5m minus E13.5m PGCs) at CpGs overlapping a binding sequence for a specific TF. The x-axis gives the average value for E14.5m PGC DNase-Hi sites, while the y-axis gives the average value for DNase-Lo sites. **B,C** Example regions showing TF accessibility and DNA methylation in preimplantation embryos at E14.5m PGC DNAse-Hi sites. CpG cov indicates the BS-seq read coverage. TF binding sequences called by fimo that overlap the peak summit in E14.5m are shown. (Related to Figure 2).

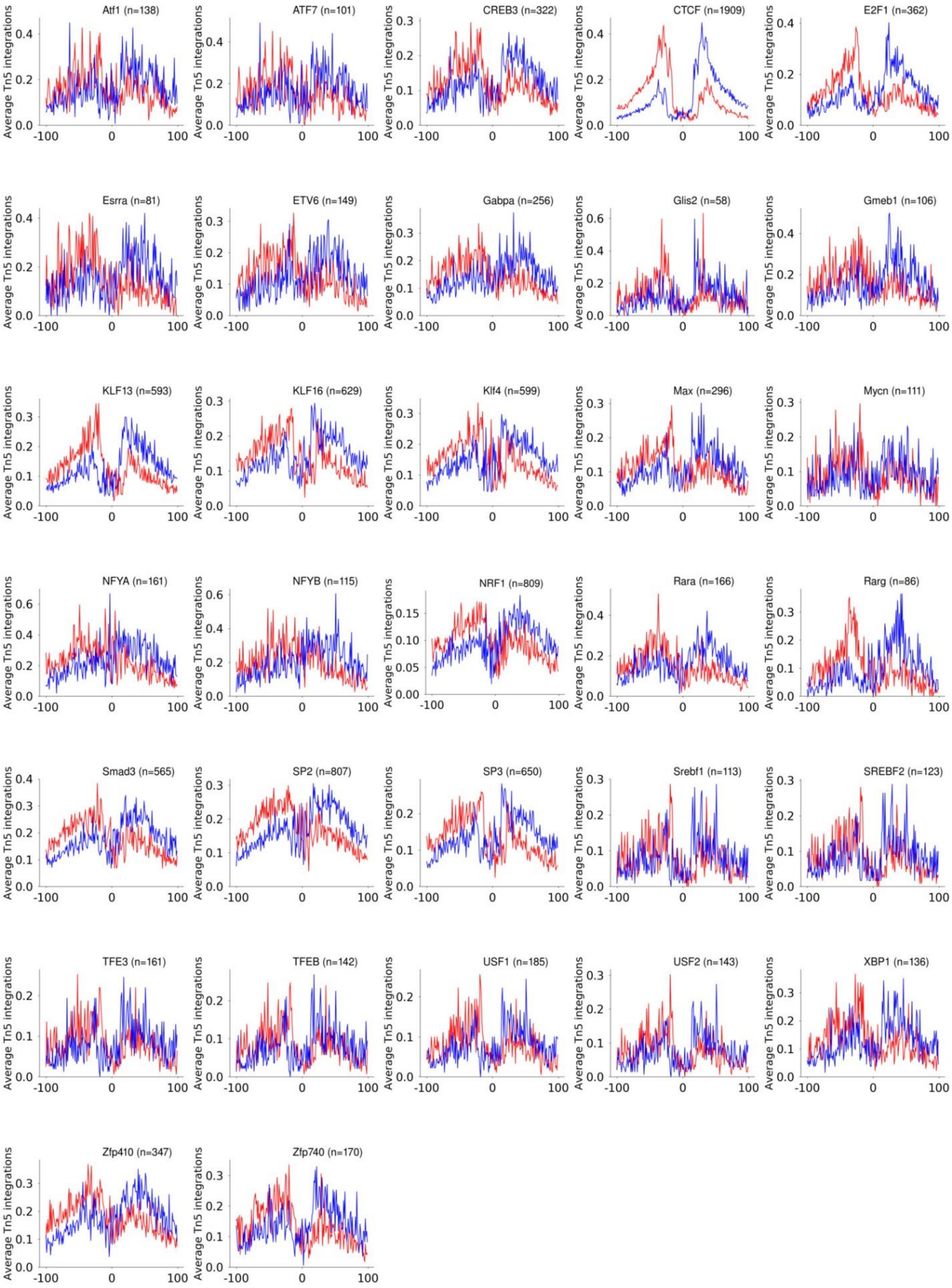
Average Tn5 integrations
Atf1 (n=138)
ATF7 (n=101)
CREB3 (n=322)
CTCF (n=1909)
E2F1 (n=362)
Esrra (n=81)
ETV6 (n=149)
Gabpa (n=256)
Glis2 (n=58)
Gmeb1 (n=106)
KLF13 (n=593)
KLF16 (n=629)
Klf4 (n=599)
Max (n=296)
Mycn (n=111)
NFYA (n=161)
NFYB (n=115)
NRF1 (n=809)
Rara (n=166)
Rarg (n=86)
Smad3 (n=565)
SP2 (n=807)
SP3 (n=650)
Srebf1 (n=113)
SREBF2 (n=123)
TFE3 (n=161)
TFEB (n=142)
USF1 (n=185)
USF2 (n=143)
XBP1 (n=136)
Zfp410 (n=347)
Zfp740 (n=170)

Figure S5. Average ATAC-seq footprint profiles in ESCs at E14.5m PGC DNAse-Hi sites containing binding sequences for the indicated TFs. (Related to Figure 2).

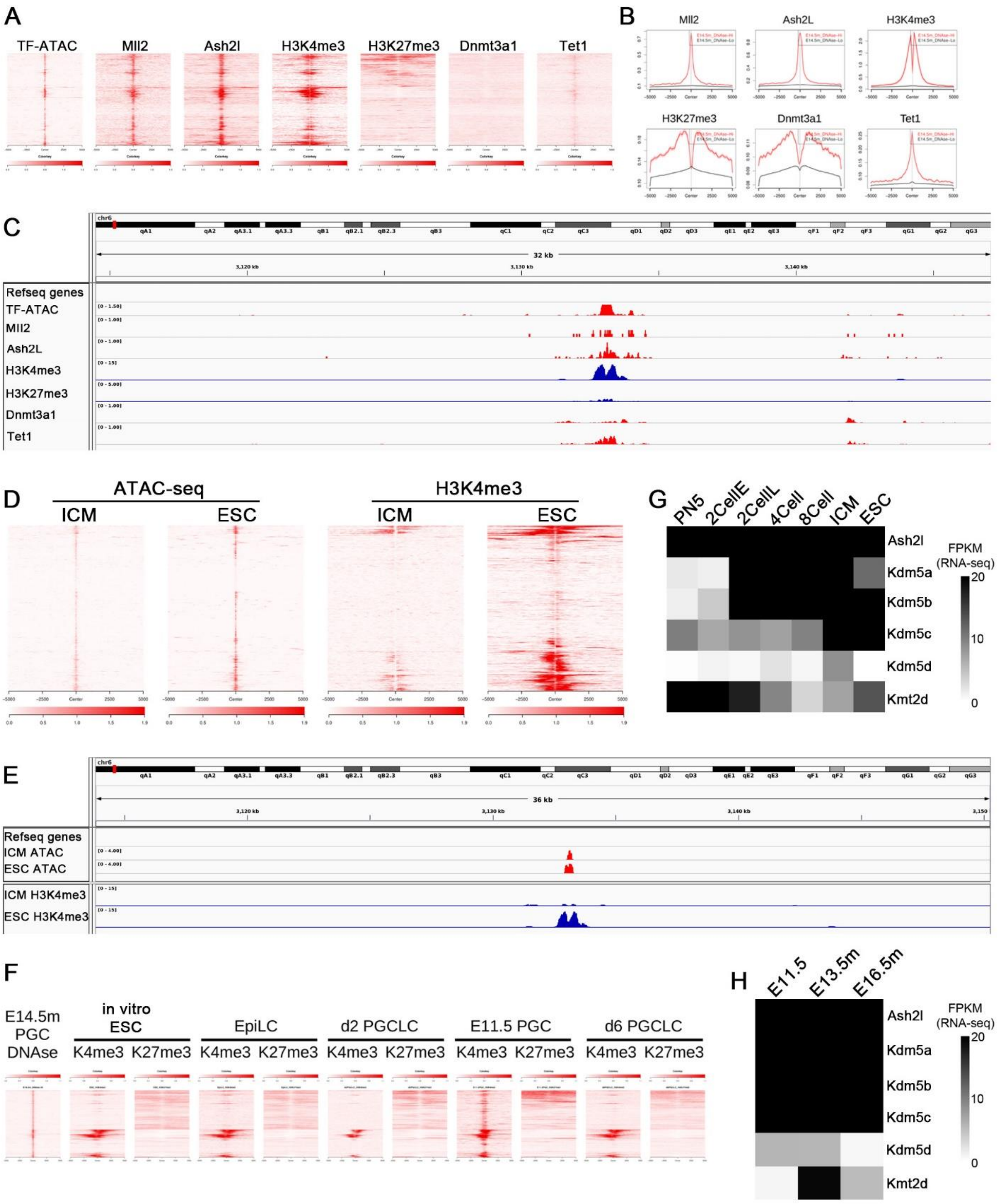

Figure S6. Co-factors at E14.5m PGC DNAse-Hi sites. **A** Heatmaps of the given ChIP-seq signal type in ESCs, centered at E14.5m PGCDNAse-Hi sites. TF-ATAC signal in ESC's is also shown for comparison. **B** Average profile of the given signal in ESCs at E14.5m PGC DNAse-Hi vs DNAse-Lo sites. **C** An example of an E14.5m PGC DNAse-Hi site, showing enrichment for the data used in A and B. **D** Heatmaps showing enrichment of TF-ATAC and H3K4me3 ChIP-

seq in ICMs vs. ESCs at E14.5m PGC DNAse-Hi sites. **E** An example showing ICM and ESC H3K4me3 ChIP-seq and TF-ATAC signal at an E14.5m PGC DNAse-Hi site. **F** Heatmaps showing H3K4me3 and H3K27me3 signal at E14.5m PGC DNAse-Hi sites for epiblast-like cells (EpiLCs), PGC-like cells 2 days and 4 days after induction (d2 PGCLCs and d4 PGCLCs, respectively), and E11.5 PGCs. The EpiLCs and PGCLCs were derived from the in vitro ESCs. **G,H** RNA-seq expression heatmap of the indicated histone demethylates and methyltransferases (G) in pre-implantation embryos and (H) PGCs. (Related to Figure 2).

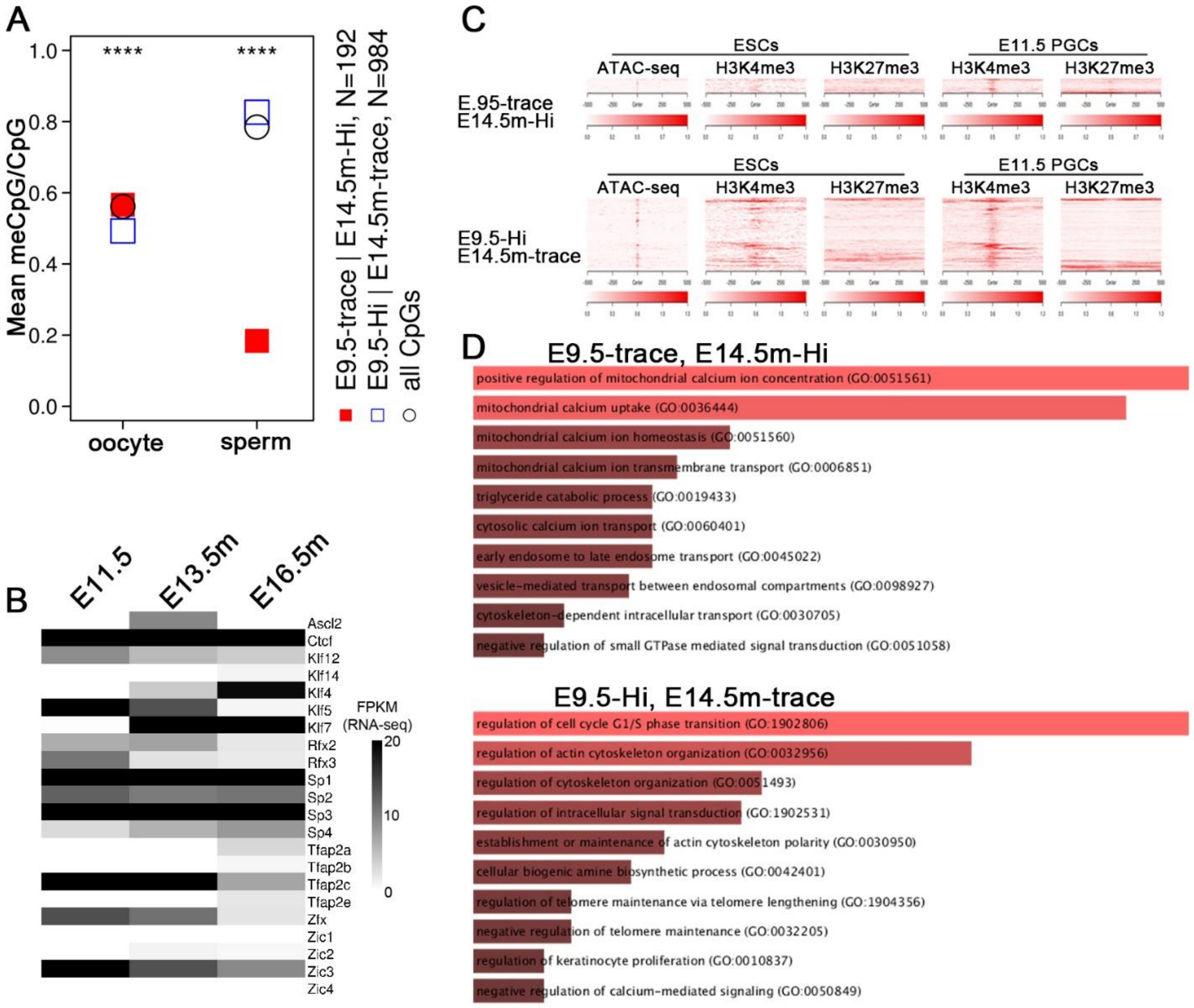

Figure S7. DNA methylation, co-factor binding, and mRNA expression at E9.5-trace, E14.5m-Hi and E9.5-Hi, E14.5m-trace sites . **A** Average DNA methylation levels as in Figure 3B, comparing sperm to oocyte methylation. P-values are by Fisher's exact test. P-val cutoffs (E9.5-trace, E14.5m-Hi vs E9.5-Hi, E14.5m-trace) *P<0.01; **P<10^{-3}; ***P<10^{-5}; ****P<10^{-10}. **B** RNA-seq of TFs enriched at E9.5-Hi, E14.5m-trace sites (Figure 3H). **C** Heatmap showing TF-ATAC in ESCs along with H3K4me3 and H3K27me3 ChIP-seq in ESCs and E11.5 PGCs, sorted by hierarchical clustering. **D** Relative Enrichr score of significantly enriched "GO Biological Process" terms for genes within 10 kb of the indicated set of loci. Only genes with FPKM>10 in E16.5m were considered for the E9.5-trace, E14.5m-Hi set, whereas only genes with FPKM>10 in E11.5 were considered for the E9.5-Hi, E14.5m-trace set. All patwhays shown have P < .05. (Related to Figure 3).

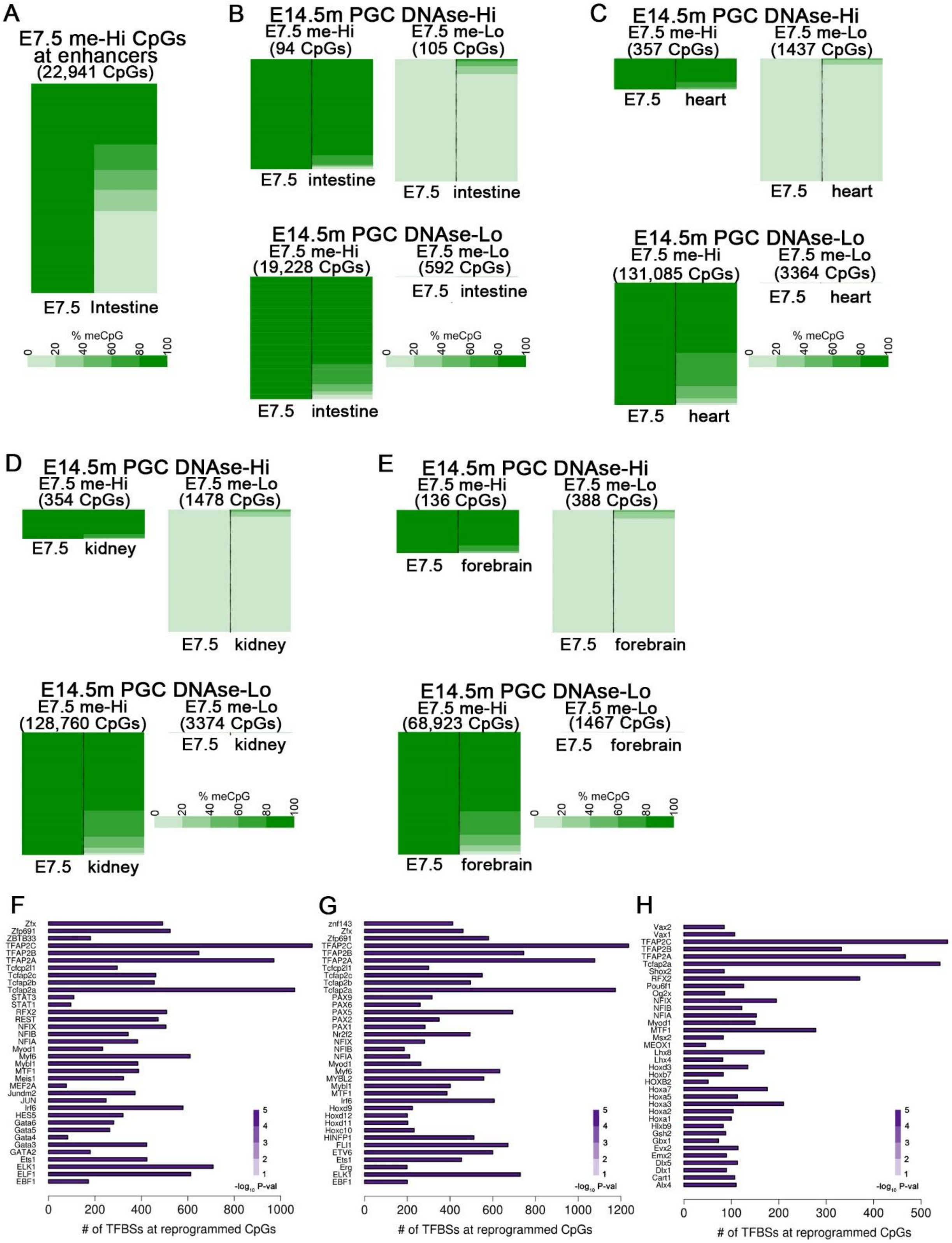
A
E7.5 me-Hi CpGs at enhancers
(22,941 CpGs)
E7.5 Intestine
% meCpG
0 20 40 60 80 100
B
E14.5m PGC DNAse-Hi
E7.5 me-Hi (94 CpGs)
E7.5 me-Lo (105 CpGs)
E7.5 intestine
E14.5m PGC DNAse-Lo
E7.5 me-Hi (19,228 CpGs)
E7.5 me-Lo (592 CpGs)
E7.5 intestine
C
E14.5m PGC DNAse-Hi
E7.5 me-Hi (357 CpGs)
E7.5 me-Lo (1437 CpGs)
E7.5 heart
E14.5m PGC DNAse-Lo
E7.5 me-Hi (131,085 CpGs)
E7.5 me-Lo (3364 CpGs)
E7.5 heart
D
E14.5m PGC DNAse-Hi
E7.5 me-Hi (354 CpGs)
E7.5 me-Lo (1478 CpGs)
E7.5 kidney
E14.5m PGC DNAse-Lo
E7.5 me-Hi (128,760 CpGs)
E7.5 me-Lo (3374 CpGs)
E7.5 kidney
E
E14.5m PGC DNAse-Hi
E7.5 me-Hi (136 CpGs)
E7.5 me-Lo (388 CpGs)
E7.5 forebrain
E14.5m PGC DNAse-Lo
E7.5 me-Hi (68,923 CpGs)
E7.5 me-Lo (1467 CpGs)
E7.5 forebrain
F
G
H
-log10 P-val
# of TFBSs at reprogrammed CpGs

Figure S8. DNA methylation maintenance from E7.5 embryos to neonatal and adult tissues. **A** Heatmap of CpG methylation percentage for all CpGs with > 80% methylation in E7.5 embryos that overlap with a published ATAC-seq peak in either embryonic, fetal, or adult intestine. The legend in A also applies to panels B-E. **B** Heatmap as described in Figure 4A, except that here only CpGs that do not overlap a published ATAC-seq peak from fetal, embryonic, or adult intestineal tissue are considered. **C-E** Heatmaps as in Figure 4A, except that instead of adult intestine, methylation is examined from BS-seq of neonatal heart (C), kidney (D), and forbrain (E). **F-H** Bar plots showing the number of motifs and the statistical significance of motif enrichment for the indicated TFs at CpGs with > 80% methylation in E7.5 embryos and < 20% methylation in neonatal heart (F), kidney (G), and forebrain (H). (Related to Figure 4).

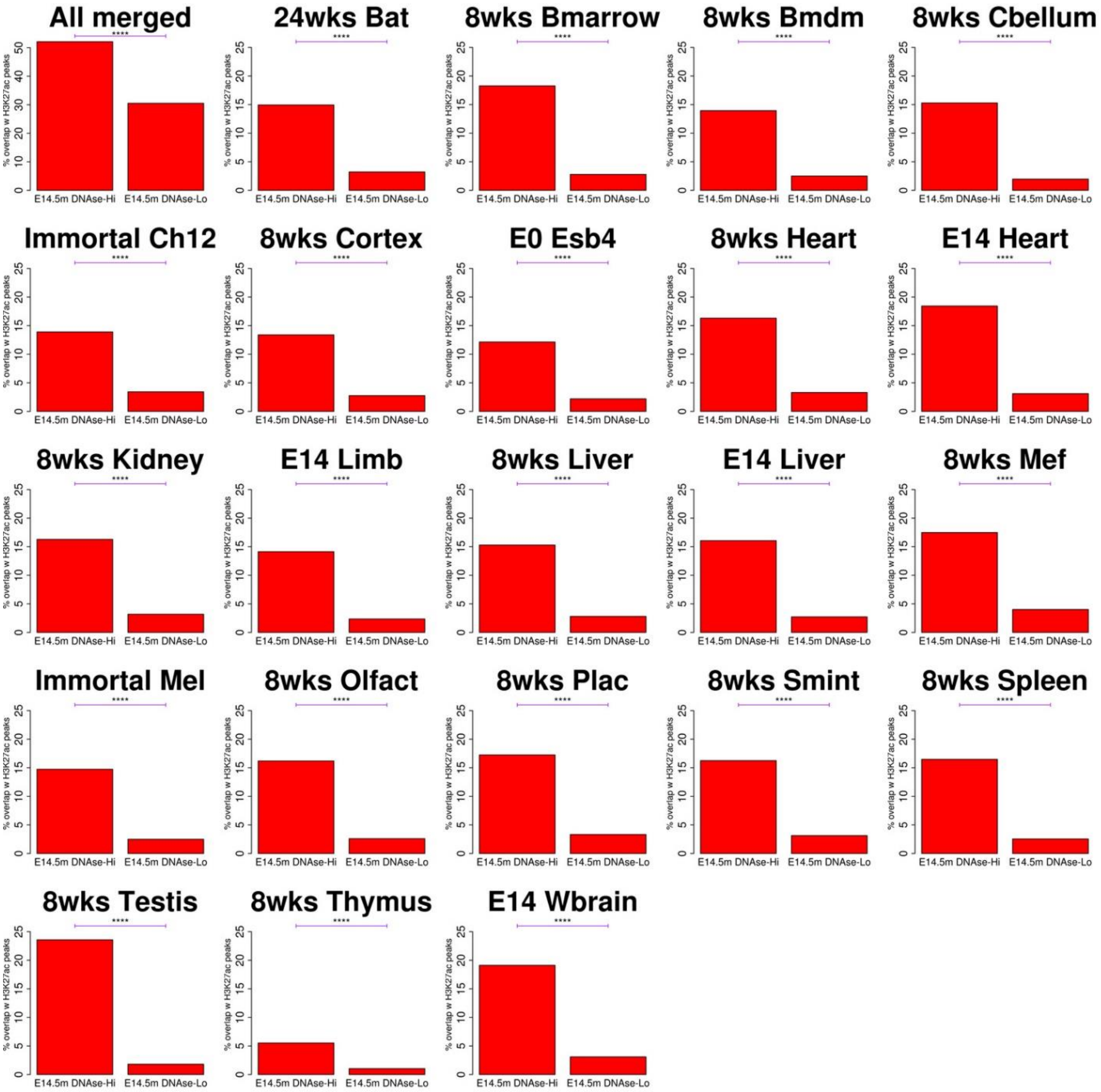

Figure S9. Overlap of E14.5m PGC DNAse-Hi and DNAse-Lo sites with H3K27ac peaks from the indicated tissue. “All merged” shows the overlap with all peaks from all samples combined. P values were determined by Fisher’s exact test, and cutoffs are as follows: *P<.01; **P<.001; ***$P<10^{-5}$; ****$P<10^{-10}$. (Related to Figure 4).

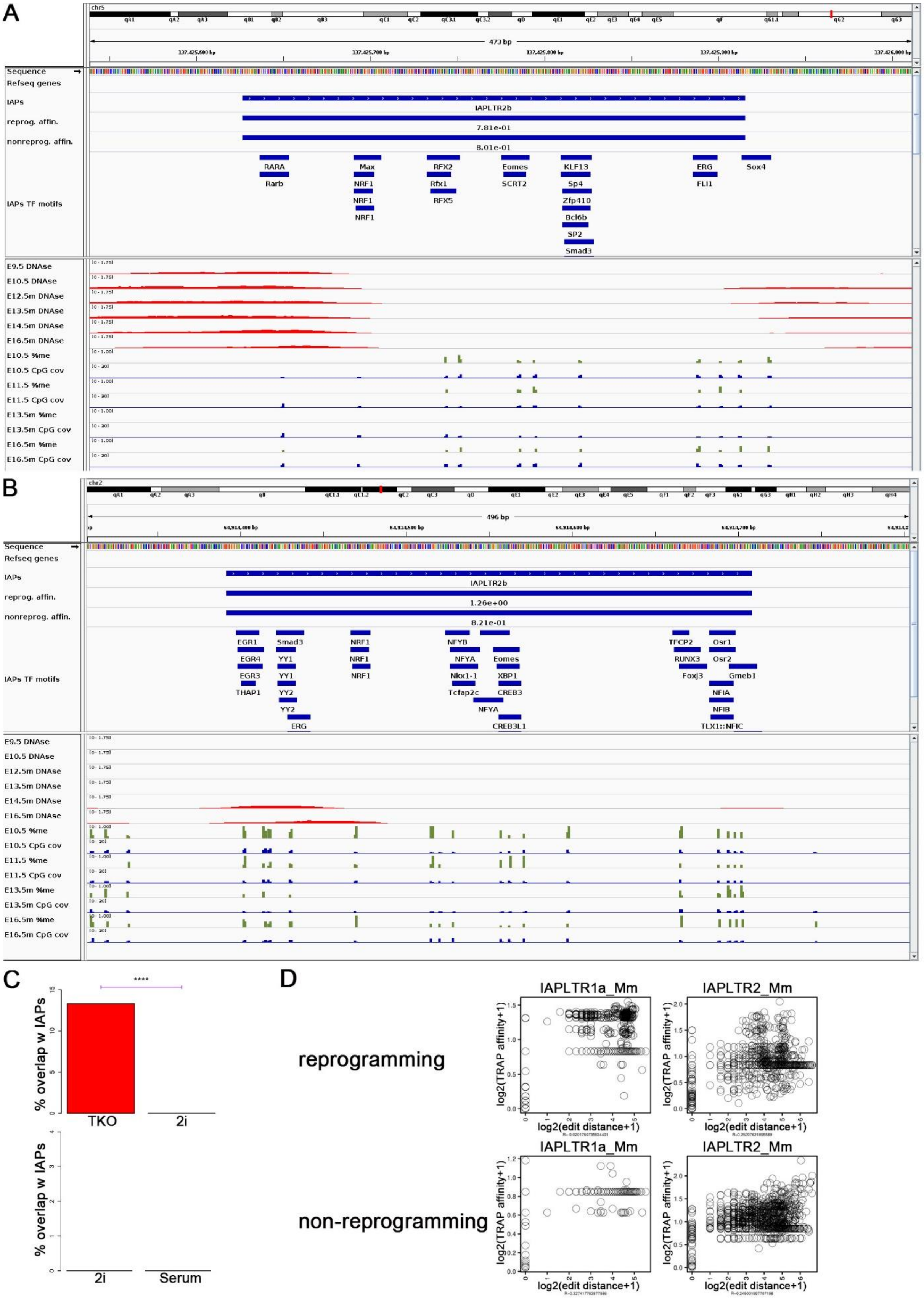
A
chr5
473 bp
Sequence
Refseq genes
IAPs
IAPLTR2b
reprog. affin.
7.81e-01
nonreprog. affin.
8.01e-01
IAPs TF motifs
RARA
Rarb
Max
NRF1
NRF1
NRF1
RFX2
Rfx1
RFX5
Eomes
SCRT2
KLF13
Sp4
Zfp410
Bcl6b
SP2
Smad3
ERG
FLI1
Sox4
E9.5 DNAse
E10.5 DNAse
E12.5m DNAse
E13.5m DNAse
E14.5m DNAse
E16.5m DNAse
E10.5 %me
E10.5 CpG cov
E11.5 %me
E11.5 CpG cov
E13.5m %me
E13.5m CpG cov
E16.5m %me
E16.5m CpG cov
B
chr2
496 bp
Sequence
Refseq genes
IAPs
IAPLTR2b
reprog. affin.
1.26e+00
nonreprog. affin.
8.21e-01
IAPs TF motifs
EGR1
EGR4
EGR3
THAP1
Smad3
YY1
YY1
YY2
YY2
ERG
NRF1
NRF1
NRF1
NFYB
NFYA
Nkx1-1
Tcfap2c
NFYA
Eomes
XBP1
CREB3
CREB3L1
TFCP2
RUNX3
Foxj3
Osr1
Osr2
Gmeb1
NFIA
NFIB
TLX1::NFIC
E9.5 DNAse
E10.5 DNAse
E12.5m DNAse
E13.5m DNAse
E14.5m DNAse
E16.5m DNAse
E10.5 %me
E10.5 CpG cov
E11.5 %me
E11.5 CpG cov
E13.5m %me
E13.5m CpG cov
E16.5m %me
E16.5m CpG cov
C
****
% overlap w IAPs
TKO
2i
% overlap w IAPs
2i
Serum
D
reprogramming
non-reprogramming
IAPLTR1a_Mm
IAPLTR2_Mm
IAPLTR1a_Mm
IAPLTR2_Mm
log2(TRAP affinity+1)
log2(edit distance+1)

Figure S10. TF binding and sequence content at IAPs. **A,B** Examples showing DNase-seq and DNA methylation levels during PGC development at IAP LTRs that have evidence of TF binding. Tracks showing the highest TRAP affinity of the E16.5m PGC putative reprogramming TFs, and of E16.5m PGC non-reprogramming TFs, at the whole IAP, are displayed under the IAP track. **C** The proportion of Nrf1 peaks overlapping with IAPs in ESC with Dnmt triple knockout (TKO), in 2i medium, and in serum. . P-vals were calculated by Fisher's Exact Test and are indicated as follows: *P<.01; **P<.001; ***P<10^{-5}; ****P<10^{-10}. **D** Scatterplots comparing TRAP affinities at IAPs to the edit distance to the consensus for IAPs of type IAPLTR1a_Mm and IAPLTR2_Mm, for putative reprogramming TFs and non-reprogramming re-methyaltion phase TFs, separately. Peasron's correlation coefficients are shown underneath each scatterplot. (Related to Figure 6).

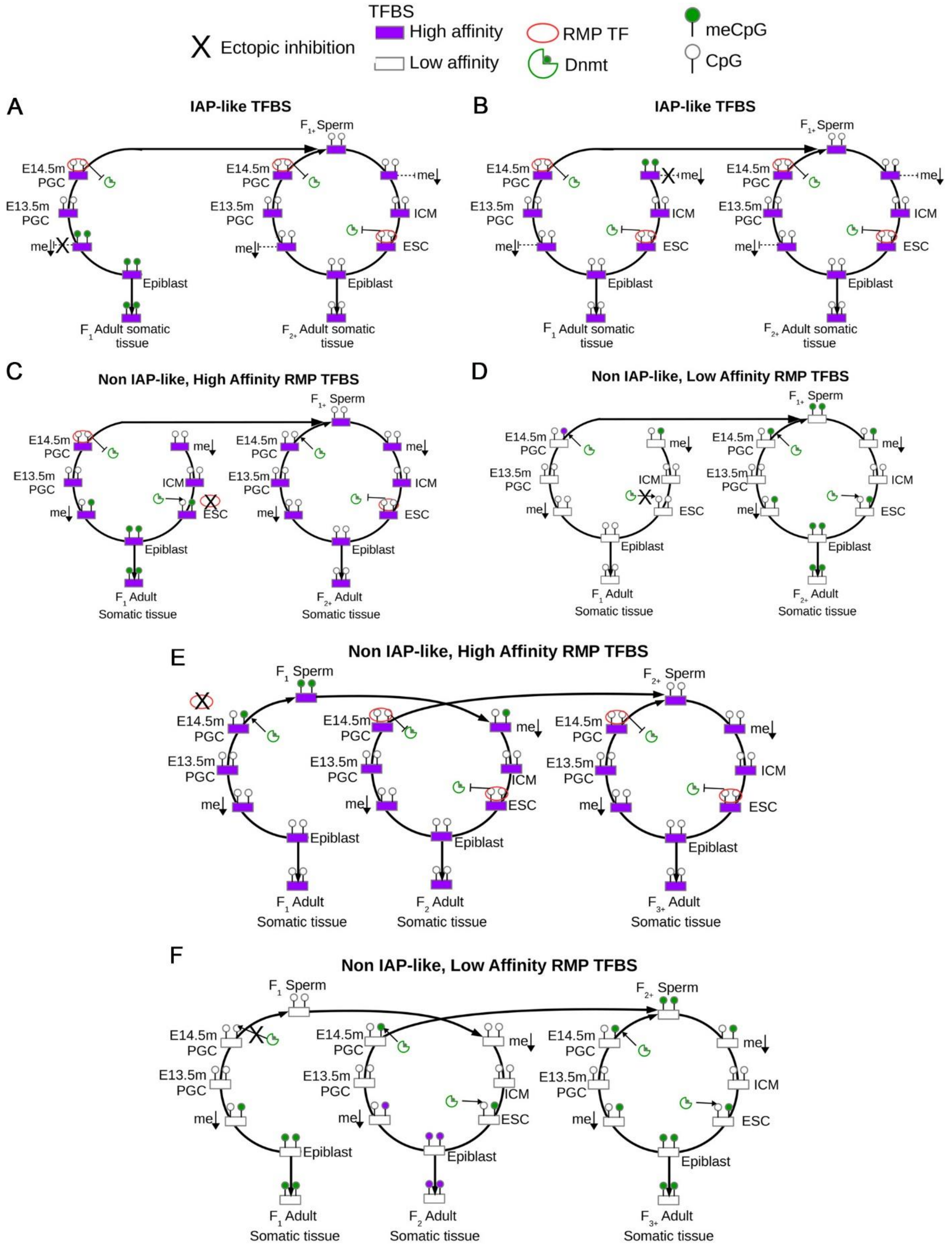
TFBS
High affinity
Low affinity
Ectopic inhibition
RMP TF
Dnmt
meCpG
CpG
A
IAP-like TFBS
B
IAP-like TFBS
C
Non IAP-like, High Affinity RMP TFBS
D
Non IAP-like, Low Affinity RMP TFBS
E
Non IAP-like, High Affinity RMP TFBS
F
Non IAP-like, Low Affinity RMP TFBS
E14.5m PGC
E13.5m PGC
ICM
ESC
Epiblast
F1+ Sperm
F1 Sperm
F2+ Sperm
F1 Adult somatic tissue
F2+ Adult somatic tissue
F1 Adult Somatic tissue
F2 Adult Somatic tissue
F2+ Adult Somatic tissue
F3+ Adult Somatic tissue
me

Figure S11. Predicted mechanisms of trans-generational and inter-generational epigenetic inheritance. **A,B** The model predicts that trans-generational epigenetic inheritance would occur by ectopically inhibiting de-methylation resistance at IAP-like loci either during PGC devolopment (A) or during pre-implantation development (B). These sites are normally methylated throughout the entire cycle (Figure 7C). Inhibition at either stage would result in trans-generational epigenetic inheritance at sites with a high affinity for re-methylation phase (RMP) TFs. However, inhibing de-methylation resistance in PGCs would result in the induced phenotype being skipped in adult somatic tissue of the F_1 offspring (A), whereas inhibiting it during pre-implantation development would result in the phenotype being present in the F_1 adult somatic tissues (B). Inhibition of de-methylation resistance might result from ectopic over-expression of a reprogramming TF such as Esrra, and possibly from ectopic changes in ncRNA expression. **C-F** The model predicts that inter-generational epigenetic inheritance would occur by ectopically inhibing re-methylation or the abundance of RMP TFs at either low or high affinity RMP TFBSs, either during the PGC RMP or the post-ICM RMP. Such inhibition could be induced by changes in ncRNA expression, histone modifications, or direct changes in RMP TF expression. Other mechanisms of inter-generational epigenetic inheritance that are independent of DNA methylation are possible wherein ncRNA from sperm are passed to the pre-implantation embryo, but this mode is not show as we are focused on the mechanistic consequences of our model. (C) Inhibiting a RMP TF in the post-ICM RMP would result in hypermethylation in the F_1 adult somatic tissue whereas these loci are normally hypomethylated in adult somatic tissue (Figure 7B). The F_1 sperm would be hypomethylated as usual because in the RMP of PGCs, TF binding at this locus would be restored. All subsequent generations would revert back to the normal behavior. (D) Inhibing post-ICM re-methylation at a low affinty RMP TFBS would result in that site being hypomethylated in F_1 adult somatic tissue whereas these loci are typically hypermethylated in adult somatic tissue (Figure 7A). F_1 sperm would revert back to being hypermethylated because re-methylation is restored to normal levels in PGCs, and all subsequent generations would have a normal phenotype. (E) Inhibiting a RMP TF binding to a high-affinity site during the PGC RMP would result in hypermethylation in the F_1 sperm whereas these loci are typically hypomethylated in sperm. The phenotype of the pre-implantation embryo would be affected, but normal binding of the TF in the post-ICM RMP would restore hypomethylation to this site in all subsequent adult sometic tissues and sperm. (F) Inhibiting re-methylation at a low-affinity RMP TFBS in the PGC RMP would result in hypomethylation of the F_1 sperm whereas these sites are typically hypermethylated in sperm (Figure 7A). The phenotype of the pre-implantation embro would be affected, but subseuently, all adult somatic tissues and sperm will be restored to the normal phenotype because of normal re-methylation in the RMPs. (Related to Figure 7).
