# Supplementary material for: Protection from DNA re-methylation by transcription factors in primordial germ cells and pre-implantation embryos can explain trans-generational epigenetic inheritance
Source: Genome Biol. 2020 May 18;21:118. doi: 10.1186/s13059-020-02036-w (PMC7236515; doi:10.1186/s13059-020-02036-w)
Supplement: Supplementary file 4 — Additional file 4. Review history. [file 13059_2020_2036_MOESM4_ESM.docx]

Review History

**First round of review**

**Reviewer 1**

**Are you able to assess all statistics in the manuscript, including the appropriateness of statistical tests used?**

Yes, and I have assessed the statistics in my report.

**Comments to author:**

This study explores the role of transcription factors as the mediators of epigenetic inheritance. They used published genome-wide BS sequencing and chromatin accessibility (DNase /ATAC-seq) data sets to study changes in DNA methylation and dynamics of distal transcription factor (TF) bound genomic loci in PGCs and embryonic development. The majority of CpGs have a similar methylation status in sperm and the E6.5 epiblast, and most of them undergo subsequent de- and re-methylation. However, CpGs associated with high accessibility and low DNA methylation in E14.5 PGCs tend to be protected from acquiring DNA methylation throughout development by TFs. In addition, they identified "reprogramming" TFs, which are specifically associated with accessible sites in early or late PGC development. In contrast, most CpGs in somatic cells retain methylation acquired in the E7.5 epiblast with only specific target sites becoming accessible and demethylated in a tissue-specific manner. IAP elements have a low affinity to reprogramming TFs, which might contribute to them evading global demethylation in the germ line. Accordingly, they suggest that acquired binding of reprogramming TFs to IAPs can result in DNA hypomethylation, which could be inherited transgenerationally. Thus, reprogramming TFs are important modulators of the DNA methylation landscape in early embryonic and PGC development. It is well established that TFs can promote both DNA de- and re-methylation through interactions with epigenetic modifiers or through sterical protection of CpGs, so the conceptual novelty in the model is somewhat limited. Also they do not include other features, e.g. H3K27me3 domains or GC content (R-loops), or provide functional evidence to support the role of the identified reprogramming TFs in development.

Major points:

The clarity of writing could be improved as the paper in parts difficult to follow, e.g. page 11 line 21 to 25, and the findings should be discussed in the context of known transcription factor effects on DNA methylation. All main figures should be ay higher resolution.

1. Based on the analysis, the authors hypothesized that binding of transcription factors to a region results in hypomethylation, and hence any gain or loss of TF binding could change DNA methylation levels, leading to inter or transgenerational epigenetic inheritance of an environmental induced phenotype. There are several gaps in this model. First, is DNA hypomethylation at enhancer the cause or the consequence of enhancer activation? How likely would variation of CpG methylation levels at enhancers cause changes in gene expression level, thus epigenetic inheritance phenotype? Second, the authors proposed that an epigenetic event that can allow ectopic TF binding to a demethylation resistant region could result in the loss of DNA methylation and epigenetic inheritance. Is there any example of this upstream "epigenetic event"? Third, it was suggested that miRNA/tRNA/piRNA could alter the transcription/translation of TFs and lead to change in DNA methylation at target loci and epigenetic inheritance. Such change in TF expression would have caused a more wide-spread effect on transcription and chromatin states, which might be more impactful than changes in DNA methylation. Finally, the role of DNA methylation as the mediator trans/intergenerational epigenetic inheritance has been questioned. For example, Shea et al (Dev Cell 2015) showed little variation in sperm DNA methylation levels in diet induced epigenetic inheritance models.

2. When comparing DNase accessible sites during PGC development, the authors discovered that DNase High and Low sites remain largely unchanged in cell cycle arrested, remethylating (E14.5/E16.5) PGCs and concluded that transcription factors remain consistently bound in these stages. Mitosis affects chromatin accessibility especially in distal transcription factor binding sites (Hsiung et al. 2015). Hence, the observed differences between E14.5/E16.5 gonocytes and earlier PGCs could be a consequence of the different proliferative state of these cells. While chromatin accessibility in conjunction with motif enrichment is indicative for the evolvement of a specific TF in the establishment or the activation of a cis-regulatory element, it is not sufficient to conclude that the same TF is constantly bound to this element in a developmental context. As ATAC-seq peaks can be found in active and poised regulatory elements (Daugherty et al., 2017), it is possible that the TF composition at cis-regulatory elements changes during the course of development without altering genomic accessibility.

3. The authors detected that DNA methylation levels were constantly low at DNase-High sites in all analysed PGC and early embryonic stages (Fig 1D, Fig2A, B). Are these DNase-High sites associated with high CpG density? It is well documented that CpG islands are predominantly unmethylated cell type independently, especially when associated with promoters. While the authors excluded a region of 2.5kb around the TSS from their analysis, CpG islands can stretch beyond this. The association of DNase-Hi sites with CpG island would be consistent with the motif enrichment analysis which resulted in the identification of many TFs that are known to regulate CpG islands e.g. Sp2, Sp3, CTCF, NRF1, GABPA etc (Hartl et al 2019). Here, the authors should provide a measure of CG content of the DNase-High sites and the average distance of these sites to the next gene.

4. In Figure 2, the authors stated that E9.5-Hi, E14.5m-trace sites that had high CpG methylation in sperm undergo rapid DNA demethylation after fertilization. However, the CpG methylation levels of these sites in oocyte have not been shown. It is possible that these sites are already demethylated in oocytes.

5. The authors need to clarify the difference between reprogramming and non-reprogramming TFs. It seems that reprogramming TFs are those TFs that are associated with re-methylation resistant sites, while non-reprogramming TFs are expressed but not associated with these sites. However, the authors focused in their study only on distal sites and excluded promoters, which leaves room for an involvement of "non-reprogramming" TF in the protection of promoters against DNA methylation.

Factors like CTCF or NRF1 have previously been reported to have impaired affinity to methylated CpGs. Hence, it is not clear whether the low DNA methylation state at CTCF and NRF1 binding sites is the cause or consequence of TF binding. The term "reprogramming TFs" might be in these cases unsubstantiated.

6. The author attributed the protection against DNA methylation in E14.5 PGCs to binding of TFs and stated that CpGs unbound by a TFs in E14.5 PGCs become remethylated. This conclusion overstates the experiential evidence shown in the manuscript. The authors showed that TF binding sites within the DNase-High sites are protected from re-methylation during PGC development. However, this does not exclude that histone modifications such as H3K27me3 or the formation of R-loops contributes to the restriction of de novo DNA methylation at cis-regulatory element during spermatogenesis. In PGCs, many developmental control genes are occupied with bivalent H3K4me3 and H3K27me3 domains, which extent beyond the core promoter. These bivalent domains are still detectable in spermatids (Lesch at al 2016). H3K27me3 domains are usually anticorrelated with DNA methylation. The authors should compare H3K27me3 occupancy in PGCs or spermatids with domains resistant against DNA re-methylation to address whether this histone modification functions in regulating DNA methylation.

7. Combining DNA methylation and DNase-seq data, the authors defined cis-regulatory elements specific to early (E9.5-Hi, E14,5m-trace) and late (E9.5-trace, E14,5m-Hi) germ cell development. While this could be an interesting and novel aspect of the paper, the conducted analysis is superficial. Here, questions that could be addressed are: Which genes are associated with these enhancers? Can these enhancers be detected in a poised state in ESCs, EpiLCs, PGCLCs or the E6.5 epiblast? Are the TFs that could potentially bind to these elements, e.g. Nrf1, expressed specifically in PGCs.

8. The TRAP analysis on E14.5 PGCs and ESCs identifies a more significant TRAP affinity score in DNase-High than at DNase-Low sites but the differences between both groups is higher in PGCs than in ESCs (page 9 line 52). The authors explained this result with the lower global 5mC level in E13.5 PGCs compared to ESCs. It is unclear how the authors come to this conclusion when E13.5 PGCs were not analyse here.

9. The authors should explain more clearly how the TRAP affinity scores were calculated and what they actually represent.

10. It is unclear why the authors chose E16.5 PGCs for the IAP analysis, as the male PGC genome is already remethylated at this time point. It would be more interesting to analyse IAPs at E13.5 in the hypermethylated genome.

11. The authors identified a positive correlation between the methylation levels of IAPs and their affinity to reprogramming TFs and suggested that high DNA methylation is a measure to prevent TFs from binding to high affinity IAPs. It has been shown before that A.) IAPs harbour TF binding sites (Shimosuga et al. 2017), and B.) depletion of DNA methylation in mouse ESCs results in expression of IAPs (Karimi et al., 2011; Matsui et al., 2010). Notably, induced reduction of 5mC in ESCs results in a surge of L1, IAP and MERVL elements expression, which is subsequently reduced through accumulation of repressive histone modifications (Walter et al., 2016). Suggesting that the cell can adapt to undesired TF targeting to transposable elements. Do reprogramming TFs bind to IAPs in DNMT depleted ESCs?

Due to the high DNA methylation levels, IAP elements have a high mutation rate. Is the accumulation of mutations in IAPs anti-correlated with the affinity to reprogramming TFs or the DNA methylation rate?

12. In Figure 6D, the IAP element only became accessible at E14.5 but the accessible region has already lost DNA methylation at E11.5-E13.5. This does not agree with the author's model that reprogramming TFs binding causes DNA demethylation.

13. It was suggested that DNA demethylation resistance of IAP involves piRNA. However, it is controversial whether piRNA is present in E13.5 PGCs.

Minor points.

1. Fig1B. A scale for the size of the plotted fragments is missing e.g. +/- 2k from the peak centre

2. Fig3H and Fig4E. It seems that some TFs are duplicated in these diagrams e.g. TFAP2c and Tcfap2c.

3. page 7 line 58: For the function of Esrra a citation is missing

4. Fig7. The depicted models are not intuitive and requires simplification. Further, part B appears truncated.

**Reviewer 2**

**Are you able to assess all statistics in the manuscript, including the appropriateness of statistical tests used?**

No, I do not feel adequately qualified to assess the statistics.

**Comments to author:**

DNA methylation is a key carrier of epigenetic information. During mammalian development, two major waves of DNA de-methylation occur: in primordial germ cells, and at the pre-implantation embryo stage (ICM of pre-implantation embryos). A substantial number of CpGs that are methylated (prior to global de-methylation) are first de-methylated and then re-methylated, whereas many CpGs that are not methylated (prior to global de-methylation) remain unmethylated even after global re-methylation. What protects these latter sites from gaining methylation? In this ms, the authors provide support for their hypothesis that "TFs may be involved in the maintenance of epigenetic information during global hypomethylation of PGCs and ESCs, ensuring that the methylation state of the majority of CpGs is preserved between generations across embryonic development". The authors propose that their TF-mediation model of epigenetic inheritance yields testable predictions for how both inter- and trans-generational epigenetic information might occur mechanistically.

This is a well written manuscript based on a comprehensive and thorough analyses of previously published data sets (BS-seq, DNAse-seq, ATAC-seq and RNA-seq). The major findings include:

-	The majority (~78%) of CpGs preserve their methylation status between sperm and E6.5 epiblasts

-	Sites with HS signal at E14.5 (male) PGCs tend to be unmethylated throughout development.

-	Methylation status of the majority of CpGs in the mammalian genome is preserved across embryonic development, and binding of TFs during the re-methylation phases is associated with the maintenance of hypomethylation across development.

-	Methylation status of the majority of CpGs at TF binding sites remains consistent between late embryonic and adult tissues (E7.5 compared to adult intestine, neonatal heart, kidney and forebrain).

-	High-affinity TF binding sites inhibit de novo DNA methylation better than low affinity sites in both PGCs and ESCs.

The concept of this work is highly interesting for a broad audience. My major criticism is that in general, HS sites are equated to transcription factor binding, which I fear is an over-simplification of the underlying biology. It is highly likely that a hypersensitive site not only indicates binding of an isolated transcription factor, but rather that this transcription factor will bring along co-factors including those with enzymatic activities. The analyses presented in this manuscript make a very convincing case for a strong correlation between HS sites and protection against DNA methylation, but they cannot discriminate between passive (blocking access for DNA methylases) and active modes (recruitment of de-methylases) of de-methylation.

It would be highly informative to devise a set of experiments to test some of the predictions of the model the authors put forward. One possibility would be to create an ectopic TF binding site in the genome, to which different versions of the TF could be recruited (full length vs versions that lack ability to bind co-factors and/or TETs), and to assess how and if these different truncation TF versions would interfere with DNA methylation. An alternative experiment would be to change a low affinity TFBS into a high affinity site. Their model would predict that this would interfere with DNA re-methylation (although even if this can be demonstrated, the question about co-factors and TETs remains). The authors may, with some justification, argue that these kind of experiments go well beyond the scope of this manuscript. In the absence of experiments to test their model, I would urge the authors to explore whether there is a way to add more bioinformatic analyses to refine the model and make predications about how re-methylation is prevented at specific sites in the genome (active vs passive).

Minor comments:

Figure 1B: what genomic distances are shown here upstream and downstream of the DNAse signal at the centre?

Figure 1B: "Some TF binding sites remain occupied throughout PGC development, whereas others are either gained or lost at specific stages." I can see this but it would help if the different categories were clearly marked in the figure.

Figure 1E: please include a y axis scale for % methylation

Figure 1E: is the example shown in a region of relatively low CpG coverage? The tracks in Figure 2D look more convincing - is the scale the same? Is the genomic region shown in Figure 1E and 2D actually the same (the genomic region window on chromosome 11 is certainly similar)?

Figure 2E: no scale - is it the same as in Figure 1G?

Page 7, line 30-32: I suggest changing "…set of regions with low DNase-seq signal in E9.5 PGCs (E9.5-trace) but with high DNase-seq in E14.5 PGCs (E14.5m-Hi), and compared them to…" to:

"…set of regions with low DNase-seq signal in E9.5 PGCs but with high DNase-seq in E14.5 PGCs (E9.5-trace, E14.5m-Hi), and compared them to…".

A cosmetic change but makes it easier to follow Figure 3.

Page 7, lines 40-41: "Both E9.5-Hi, E14.5m-trace and E9.5-trace, E14.5m-Hi show no discernible pattern of TF binding during pre-implantation development and in sperm (Figure 3C)."

I tend to agree with this conclusion for E9.5-trace, E14.5m-Hi (upper panels), but there appear to be quite pronounced changes for E9.5-Hi, E14.5m-trace (lower panels).

Figure 3C: are the numbers correct here, n=192 for the upper panels and n=984 for the lower panels? This is hard to verify for based on the figure for me but my impression is that more sites are shown in the upper panels compared to the lower panels?

Figure 4E: are both mouse and human TFs listed here? If yes, why (as dynamic methylation of the mouse genome during development is the focus of this study)? If not, why is the nomenclature not consistent (for example HOXD13 but Hoxd12)?

Figure 5G legend: "Example showing the Nrf1 ChiP-seq (minor: should be ChIP-seq) and DNA methylation levels during and after growth in 2i medium at a Nrf1 peak with relatively high TRAP affinity."

Is this correct? My interpretation of the figure is that two different Nrf1 binding sites are shown, presumably one with low TRAP affinity (gaining DNA methylation and losing Nrf1 binding) in the upper panels and a different one with high TRAP affinity (sustained Nrf1 binding and no changes in DNA me) in the lower panels?

Several typos in the discussion on page 13:

-	Line 34: "…miRNA's…" (should be miRNAs?)

-	Lines 37/38: "…in pre-implantation embryos in studies in studies…"

-	Lines 38/39: "…in a low protein diet-induced trans-generational epiphenotypes." Omit 'a'?

-	Lines 39/40: "These studies also observed that the level on several piRNAs were altered in sperm…" should be "…was altered" or "…levels…were altered"?

The design of figure 7 should be improved:

-	Why are there three colours for CpG sites? Green for methylated, light blue for unmethylated and white for?

-	The symbol for DNA demethylase should be consistent either a grey or a green diamond

-	IAPs should be represented as a box with dashed lines in the legend

-	'2Cell morula' is tilted

**Authors Response**

**Point-by-point responses to the reviewers’ comments:**

*We would like to thank both reviewers for their careful reading of the manuscript and their constructive remarks, which have helped us improve the quality of the paper substantially. We have addressed every issue raised by the reviewers and below is a point by point description of the changes we have made:*

Reviewer 1

Major points:

“The clarity of writing could be improved as the paper in parts difficult to follow, e.g. page 11 line 21 to 25, and the findings should be discussed in the context of known transcription factor effects on DNA methylation. All main figures should be ay higher resolution”.

*We have read through the text and tried to improve the readability. We have included additional discussion of our results in the context of known transcription factor effects on DNA methylation (e.g. page 16, line 30 -page 17, line 2). We have also increased the resolution and size of the main figures. All main figures submitted with the revised manuscript have a native resolution of 300 dpi.*

1. “Based on the analysis, the authors hypothesized that binding of transcription factors to a region results in hypomethylation, and hence any gain or loss of TF binding could change DNA methylation levels, leading to inter or transgenerational epigenetic inheritance of an environmental induced phenotype. There are several gaps in this model. First, is DNA hypomethylation at enhancer the cause or the consequence of enhancer activation? How likely would variation of CpG methylation levels at enhancers cause changes in gene expression level, thus epigenetic inheritance phenotype? Second, the authors proposed that an epigenetic event that can allow ectopic TF binding to a demethylation resistant region could result in the loss of DNA methylation and epigenetic inheritance. Is there any example of this upstream "epigenetic event"? Third, it was suggested that miRNA/tRNA/piRNA could alter the transcription/translation of TFs and lead to change in DNA methylation at target loci and epigenetic inheritance. Such change in TF expression would have caused a more wide-spread effect on transcription and chromatin states, which might be more impactful than changes in DNA methylation. Finally, the role of DNA methylation as the mediator trans/intergenerational epigenetic inheritance has been questioned. For example, Shea et al (Dev Cell 2015) showed little variation in sperm DNA methylation levels in diet induced epigenetic inheritance models”.

*The reviewer raises several important issues that we have tried to address in the revised version of the manuscript. We acknowledge in the manuscript that the work of Shea et al shows that, in this specific case, the authors did not find changes in DNA methylation that correlate with the transmission of the phenotype. The main point of our manuscript is to show that analyses of DNA methylation and TF occupancy show a strong correlation between the presence of bound TFs at the time when DNA is de-methylated in PGCs and pre-implantation development and the failure of the bound sites to become re-methylated. We then suggest that environmental factors able to disrupt TF occupancy may alter DNA methylation levels and result in transmissible epiphenotypes. It is possible that endocrine disrupting chemicals, which act through nuclear hormone receptors and are therefore good candidates to disrupt TF distribution, act through this mechanism. The idea is that, once a specific binding site is not methylated early in development, this site can bind TFs in differentiated tissues and activate gene expression. In order to address the issue raised about the cause-effect relationship between hypomethylated DNA and enhancer activation, and the likelihood of hypomethylation at DNAse-Hi sites to cause changes in gene expression, we examined H3K27ac peaks from 22 mouse tissue from ENCODE, and found that in every tissue, a significantly larger proportion of the E14.5m DNAse-Hi sites overlap with H3K27ac peaks than do the DNAse-Lo sites. On page 11, line 41 – page 12, line 25, we discuss the implications of this result for the cause-effect relationship between hypomethylated DNA and activation of enhancers, and for the likelihood that hypomethylation due to TF binding E14.5m PGCs and ESCs will result in changes in gene expression. Several lines of evidence suggest that ectopic overexpression of either Esrra or Esrrb during PGC or embryonic development may result in a trans-generational change in phenotype via de-methylation at IAPs. We have added a summary of this evidence to the Discussion (page 16, line 15-24). We agree with the reviewer that changes in transcription or translation of TFs will have widespread effects on transcription and chromatin state, which might be more impactful than changes in DNA methylation in the immediate term. However, our model predicts that the only changes that will be maintained trans-generationally are decreases in methylation at IAPs (and downstream effects of that). We have described these points in greater detail in the Discussion (page 17, lines 25-38). Finally, it is important to note that our model predicts that changes in DNA methylation are only required for trans-generational changes in phenotype, and not inter-generational changes. The study by Shea et al. only observed inter-generational changes, so their work is consistent with the predictions of our model. Our model therefore provides an explanation for why many effects are only inter-generational whereas only some are trans-generational. We have added a discussion of this on page 17, line 39- page 18, line 18. We have also added a supplementary figure (Additional file 1: Figure S11) to explicitly show specific mechanisms of inter- and trans-generational epigenetic inheritance predicted by our model.*

2. “When comparing DNase accessible sites during PGC development, the authors discovered that DNase High and Low sites remain largely unchanged in cell cycle arrested, remethylating (E14.5/E16.5) PGCs and concluded that transcription factors remain consistently bound in these stages. Mitosis affects chromatin accessibility especially in distal transcription factor binding sites (Hsiung et al. 2015). Hence, the observed differences between E14.5/E16.5 gonocytes and earlier PGCs could be a consequence of the different proliferative state of these cells. While chromatin accessibility in conjunction with motif enrichment is indicative for the evolvement of a specific TF in the establishment or the activation of a cis-regulatory element, it is not sufficient to conclude that the same TF is constantly bound to this element in a developmental context. As ATAC-seq peaks can be found in active and poised regulatory elements (Daugherty et al., 2017), it is possible that the TF composition at cis-regulatory elements changes during the course of development without altering genomic accessibility”.

*We agree with this point and we have now cited the studies mentioned by the reviewer in the text and have considered them in our interpretation of the results on page 6, lines 8-20. Given these studies, we no longer interpret the persistence in accessibility between E14.5m and E16.5m to mean that the same TF remains persistently bound at each site. Importantly, this does not affect our model, since as long as some factor remains present to inhibit de-novo methylation during the global remethylation phase, the site will remain hypomethylated. The specific factors can change as long as there is at least one factor present at all time capable of protecting against methylation, and the site will remain hypomethylated. We have also stated explicitly the possibility that the DNAse-Hi sites may remain bound by TFs during the mitotic phase of PGC development despite dynamic changes in DNAse accessibility.*

3. “The authors detected that DNA methylation levels were constantly low at DNase-High sites in all analysed PGC and early embryonic stages (Fig 1D, Fig2A, B). Are these DNase-High sites associated with high CpG density? It is well documented that CpG islands are predominantly unmethylated cell type independently, especially when associated with promoters. While the authors excluded a region of 2.5kb around the TSS from their analysis, CpG islands can stretch beyond this. The association of DNase-Hi sites with CpG island would be consistent with the motif enrichment analysis which resulted in the identification of many TFs that are known to regulate CpG islands e.g. Sp2, Sp3, CTCF, NRF1, GABPA etc (Hartl et al 2019). Here, the authors should provide a measure of CG content of the DNase-High sites and the average distance of these sites to the next gene”.

*We agree with this important point and we have now created boxplots to compare the CpG density of the DNAse-Hi sites to DNAse-Lo sites within CpG islands and not within CpG islands. The CpG density is significantly lower to that of DNAse-Lo sites within CpG islands and is more similar to DNAse-Lo sites not at CpG islands. We also generated a histogram of the distances from DNAse-Hi sites to the nearest gene and found that most were either intragenic or nearby gene bodies. The three plots mentioned here have been implemented as Figures S2A-C. We also added a short paragraph starting at page 6, line 36 of the text to describe these results.*

4. “In Figure 2, the authors stated that E9.5-Hi, E14.5m-trace sites that had high CpG methylation in sperm undergo rapid DNA demethylation after fertilization. However, the CpG methylation levels of these sites in oocyte have not been shown. It is possible that these sites are already demethylated in oocytes”.

*We have now included a plot of the methylation levels in oocytes at E9.5-Hi, E14.5m-trace sites to the supplementary material (Additional file 1: Figure S7A). Indeed, the methylation levels were lower in oocytes than sperm, but not enough to account for the large drop seen after fertilization: the levels are ~80% in sperm and ~50% in oocytes (~65% gamete average), but only ~20% at the 2Cell stage. We have discussed this in the text (page 9, lines 22-35).*

5. “The authors need to clarify the difference between reprogramming and non-reprogramming TFs. It seems that reprogramming TFs are those TFs that are associated with re-methylation resistant sites, while non-reprogramming TFs are expressed but not associated with these sites. However, the authors focused in their study only on distal sites and excluded promoters, which leaves room for an involvement of "non-reprogramming" TF in the protection of promoters against DNA methylation. Factors like CTCF or NRF1 have previously been reported to have impaired affinity to methylated CpGs. Hence, it is not clear whether the low DNA methylation state at CTCF and NRF1 binding sites is the cause or consequence of TF binding. The term "reprogramming TFs" might be in these cases unsubstantiated.”

*We have added a definition of what we mean by “reprogramming” in this manuscript (page 8, line 43 – page 9, line 5). We found that all bound TFs are associated with protection from de-novo methylation during the re-methylation phases, regardless of whether they are reprogramming or non-reprogramming TFs. This is most clearly illustrated in Figure 1F, where we examine all re-methylation phase TFs separately regardless of whether they are reprogramming or non-reprogramming. The majority of CpGs follow the same pattern of either 1) being methylated in the epiblast, sperm, and adult somatic tissue despite being de-methylated in PGCs and pre-implantation embryos, or 2) remaining hypomethylated in the epiblast, sperm, adult somatic tissues, PGCs, and pre-implantation embryos. We therefore think it is reasonable to define “reprogramming” in our manuscript as breaking the canonical pattern of 1) or 2) above. We address the issue of cause vs. consequence of TF binding and hypomethylation more extensively below and in the revised version of the manuscript. Briefly, Stadler et al. (2011, Nature) showed direct evidence that the binding of CTCF can promote local de-methylation of nearby CpGs. Adachi et al. (2018, Cell Stem Cell) showed that Esrrb can bind to methylated DNA and promote local de-methylation. Ctcf and Esrrb therefore represent TFs with experimentally-validated potential for reprogramming according to our definition.*

6. “The author attributed the protection against DNA methylation in E14.5 PGCs to binding of TFs and stated that CpGs unbound by a TFs in E14.5 PGCs become remethylated. This conclusion overstates the experiential evidence shown in the manuscript. The authors showed that TF binding sites within the DNase-High sites are protected from re-methylation during PGC development. However, this does not exclude that histone modifications such as H3K27me3 or the formation of R-loops contributes to the restriction of de novo DNA methylation at cis-regulatory element during spermatogenesis. In PGCs, many developmental control genes are occupied with bivalent H3K4me3 and H3K27me3 domains, which extent beyond the core promoter. These bivalent domains are still detectable in spermatids (Lesch at al 2016). H3K27me3 domains are usually anticorrelated with DNA methylation. The authors should compare H3K27me3 occupancy in PGCs or spermatids with domains resistant against DNA re-methylation to address whether this histone modification functions in regulating DNA methylation”.

*We agree with the reviewer that our results alone cannot determine whether TF binding plays a direct or indirect role in protection from DNA methylation and did not mean to imply this in the manuscript. We have therefore changed the wording to make this clearer. We have added a section and supplementary figure where we examine H3K4me3 and H3K27me3 in regions flanking E14.5m PGC DNAse-Hi sites. We observed enrichment of H3K4me3 flanking all E14.5m PGC DNAse-Hi sites in ESCs and E11.5 PGCs, only some of which were also occupied by H3K27me3. We also observed an enrichment of Mll2 and Ash2l at these sites in ESCs. Importantly, we observed no such enrichment of H3K4me3 in the ICM even though ICM ATAC-seq demonstrates that many of these sites are already bound by a TF, suggesting that TF binding precedes the establishment of H3K4me3 in these regions. In addition, we observed an enrichment of Tet1 at all of these sites in ESCs. Taken together, our results suggest that it is likely that multiple, redundant mechanisms of protection from DNA methylation exist at these sites, i.e. TFs play both a direct and indirect role in protecting from methylation. We summarize this in the Discussion of our revised manuscript (starting page 18, line 19).*

7. “Combining DNA methylation and DNase-seq data, the authors defined cis-regulatory elements specific to early (E9.5-Hi, E14,5m-trace) and late (E9.5-trace, E14,5m-Hi) germ cell development. While this could be an interesting and novel aspect of the paper, the conducted analysis is superficial. Here, questions that could be addressed are: Which genes are associated with these enhancers? Can these enhancers be detected in a poised state in ESCs, EpiLCs, PGCLCs or the E6.5 epiblast? Are the TFs that could potentially bind to these elements, e.g. Nrf1, expressed specifically in PGCs”.

*We have followed the suggestions of the reviewer and we have now identified genes associated with these enhancers based on RNA-seq and genomic distance to the enhancer site. We have also performed an enrichment analysis of these genes and included this information in Additional file 1: Figure S7. We identified a subset of these sites in a poised state in ESCs and E11.5 PGCs based on H3K27me3 ChIP-seq. Epiblast-like cells and PGC-like cells in vitro cell models did no fully recapitulate comparable in vivo signal (Additional file 1: Figure S6A vs. F) at E14.5m DNAse-Hi sites, suggesting that only a subset of these sites are bound in the Epi-like and PGC-like samples. We therefore did not examine the Epi-like and PGC-like data in this context. Finally, we have indicated in the text and Additional file 1: Figure S7 which of these TFs are expressed in specific stages of PGC development. Although these reprogrammable sites were not the main focus of our manuscript, we recognize they may be of interest to various researchers and hope we have now provided enough details about these sites to assist those interested in conducting follow-up studies.*

8. “The TRAP analysis on E14.5 PGCs and ESCs identifies a more significant TRAP affinity score in DNase-High than at DNase-Low sites but the differences between both groups is higher in PGCs than in ESCs (page 9 line 52). The authors explained this result with the lower global 5mC level in E13.5 PGCs compared to ESCs. It is unclear how the authors come to this conclusion when E13.5 PGCs were not analysed here”.

*We thank the reviewer for pointing out this inconsistency. We mistakenly wrote in the original submission that the global methylation of E13.5m PGCs is lower than that of ESCs, whereas we meant that the global methylation of E13.5m PGCs is lower than that of the ICM. We have corrected this in the text (page 13, line 5). This makes it likely that, early on in the re-methylation phase, global methylation will be higher in ESCs than PGCs. TRAP affinities are calculated based on pure sequence content. Therefore, in cells with higher global methylation, the TRAP affinity will be less reflective of the actual affinity for methylation-sensitive TFs than in cells with lower global methylation. Thus, it is likely that the TRAP affinity is more accurate for PGCs than ESCs due to the lower methylation in PGCs. We have added an explanation of this in the text (page 12, lines 7-11).*

9. “The authors should explain more clearly how the TRAP affinity scores were calculated and what they actually represent”.

*We have added a clearer explanation of how TRAP affinity scores are calculated and what they actually represent in the text (page 12, lines 36-41).*

10. “It is unclear why the authors chose E16.5 PGCs for the IAP analysis, as the male PGC genome is already remethylated at this time point. It would be more interesting to analyse IAPs at E13.5 in the hypermethylated genome”.

*We chose E16.5m PGCs for the TF analysis at IAPs in Figure 6 because we were interested in determining if a TF bound during the global re-methylation phase would result in protection from re-methylation at IAPs as it does at non-IAP sites. According to our model, it is possible for a TF binding site to remain unbound until the start of global re-methylation and still be protected from re-methylation. This is observed at the IAP in Figure 6D, where DNAse-seq signal occurs in E14.5m and E16.5m, but not E13.5m. RNA-seq data are available only for E16.5m and not E14.5m, hence the reason we focused solely on E16.5m.*

11. “The authors identified a positive correlation between the methylation levels of IAPs and their affinity to reprogramming TFs and suggested that high DNA methylation is a measure to prevent TFs from binding to high affinity IAPs. It has been shown before that A.) IAPs harbour TF binding sites (Shimosuga et al. 2017), and B.) depletion of DNA methylation in mouse ESCs results in expression of IAPs (Karimi et al., 2011; Matsui et al., 2010). Notably, induced reduction of 5mC in ESCs results in a surge of L1, IAP and MERVL elements expression, which is subsequently reduced through accumulation of repressive histone modifications (Walter et al., 2016). Suggesting that the cell can adapt to undesired TF targeting to transposable elements. Do reprogramming TFs bind to IAPs in DNMT depleted ESCs? Due to the high DNA methylation levels, IAP elements have a high mutation rate. Is the accumulation of mutations in IAPs anti-correlated with the affinity to reprogramming TFs or the DNA methylation rate?”.

*We thank the reviewer for this information and suggestions. We have now cited the references mentioned by the reviewer in the text and discussed the relevant findings (page 14, line 6; page 16, line 33 – page 17, line 2). We observed binding of the reprogramming TF Nrf1 in Dnmt TKO ESCs but not in ESCs in 2i medium and found little correlation between the accumulation of mutations within IAP LTRs and the overall affinity for reprogramming TFs at those IAPs. We have incorporated this information in the manuscript. The implications of these results on our model, and the associated figures, are discussed on page 15, lines 14-27.*

12. “In Figure 6D, the IAP element only became accessible at E14.5 but the accessible region has already lost DNA methylation at E11.5-E13.5. This does not agree with the author's model that reprogramming TFs binding causes DNA demethylation”.

*We apologize for this misunderstanding, which is due to our failure to make clear what we understand by reprograming transcription factors in the original version of the manuscript. Our model is that the binding of any TF during the re-methylation phase (after E13.5) will result in protection against de-novo methylation. According to this, once a site is protected from de-novo methylation, the hypomethylated state will be maintained in sperm and passed on to the fertilized embryo. Hence, the model predicts that the site will remain hypomethylated in E9.5-E13.5m of the next generation regardless of whether or not TF binding occurs at that time. In this context, the example shown in Figure 6D is therefore consistent with this view.*

13. “It was suggested that DNA demethylation resistance of IAP involves piRNA. However, it is controversial whether piRNA is present in E13.5 PGCs”.

*We agree with the reviewer. We have now mentioned the lack of understanding of the activity of piRNAs during early PGC development and have added a relevant reference (page 17, lines 3-5). We no longer assert piRNAs as the likely mechanism for de-methylation resistance, as it seems likely that some additional, possibly redundant mechanism is active at this time.*

Minor points:

1. “Fig1B. A scale for the size of the plotted fragments is missing e.g. +/- 2k from the peak centre”.

*Thank you for pointing out this oversight. We have now added a scale to indicate the size of the plotted regions for Figs. 1A and 2B.*

2. “Fig3H and Fig4E. It seems that some TFs are duplicated in these diagrams e.g. TFAP2c and Tcfap2c”.

*We have removed redundant occurrences of genes from these diagrams.*

3. “page 7 line 58: For the function of Esrra a citation is missing”.

*We added two references addressing the function of Esrra on page 9, line 39 (previously page 7, line 58).*

4. “Fig7. The depicted models are not intuitive and requires simplification. Further, part B appears truncated”.

*We have re-drawn and simplified the models in Fig. 7 and ensured that it is no longer truncated.*

Reviewer 2

“My major criticism is that in general, HS sites are equated to transcription factor binding, which I fear is an over-simplification of the underlying biology. It is highly likely that a hypersensitive site not only indicates binding of an isolated transcription factor, but rather that this transcription factor will bring along co-factors including those with enzymatic activities. The analyses presented in this manuscript make a very convincing case for a strong correlation between HS sites and protection against DNA methylation, but they cannot discriminate between passive (blocking access for DNA methylases) and active modes (recruitment of de-methylases) of de-methylation”.

*We fully agree that cofactors recruited by the original TF are likely to be involved in the establishment and/or maintenance of the methylated state of specific sequences. To address the concerns of the reviewer, we have now including results from analyses of potential co-factors that may be involved in the protection from DNA methylation at E14.5m PGC DNAse-Hi sites. It has been shown previously that H3K4me3 represses de-novo DNA methylation because Dnmt3l binds exclusively to unmethylated H3K4. Although H3K4me3 is typically found at promoters rather than enhancers, it has been shown that distal H3K4me3 domains associated with hypomethylated DNA are critical in the transition from oocytes to zygotes. We wondered whether such distal H3K4me3 domains might play a role in blocking de-novo methylation at the TF binding sites identified in our study as being bound during the global DNA re-methylation phases in PGCs and ESCs. To test this, we examined published ChIP-seq data for H3K4me3 as well as Mll2 and Ash2l, factors involved in the methylation of H3K4. We also examined ChIP-seq of Tet1 at the same regions. We observed enrichment of H3K4me3 flanking all E14.5m PGC DNAse-Hi sites in ESCs and E11.5 PGCs. We also observed an enrichment of Mll2 and Ash2l at these sites in ESCs. Importantly, we observed no such enrichment of H3K4me3 in the ICM even though ICM ATAC-seq demonstrates that many of these sites are already bound by a TF, suggesting that TF binding precedes the establishment of H3K4me3 in these regions. In addition, we observed an enrichment of Tet1 at all of these sites in ESCs. Nonetheless a few studies, now cited in the discussion, show convincing evidence that the binding of some TFs can directly protect DNA from being methylated. Taken together, our results suggest it is likely that multiple, redundant mechanisms of protection from DNA methylation exist at these sites, i.e. TFs play both a direct and indirect role in protecting from methylation. We summarize this in the Discussion of the revised manuscript starting at page 18, line 19.*

“It would be highly informative to devise a set of experiments to test some of the predictions of the model the authors put forward. One possibility would be to create an ectopic TF binding site in the genome, to which different versions of the TF could be recruited (full length vs versions that lack ability to bind co-factors and/or TETs), and to assess how and if these different truncation TF versions would interfere with DNA methylation. An alternative experiment would be to change a low affinity TFBS into a high affinity site. Their model would predict that this would interfere with DNA re-methylation (although even if this can be demonstrated, the question about co-factors and TETs remains). The authors may, with some justification, argue that these kind of experiments go well beyond the scope of this manuscript. In the absence of experiments to test their model, I would urge the authors to explore whether there is a way to add more bioinformatic analyses to refine the model and make predications about how re-methylation is prevented at specific sites in the genome (active vs passive)”.

*We fully agree with the reviewer that the proposed experiments would strengthen the conclusions of the manuscript. However, we hope the reviewer recognizes that these experiments would take a very long period of time to complete, even if restrictions to work in the lab are lifted within the next few months. However, we have followed the suggestions of the reviewers by including additional analyses of existing data as described in the previous point. The results from these analyses clearly validate the issues raised by the reviewer and we have incorporated these observations in the revised version of the manuscript, making clear that other co-factors are involved in the establishment and/or maintenance of methylated states.*

Minor comments:

“Figure 1B: what genomic distances are shown here upstream and downstream of the DNAse signal at the centre?”.

*We have now added a scale to indicate the distances up- and down-stream of the DNAse signal at the center.*

“Figure 1B: "Some TF binding sites remain occupied throughout PGC development, whereas others are either gained or lost at specific stages." I can see this but it would help if the different categories were clearly marked in the figure”.

*We added vertical black lines at the right of the heatmap to indicate regions with persistently high DNAse signal across all stages of PGC development and added a description of this in the figure legend.*

“Figure 1E: please include a y axis scale for % methylation”.

*y-axis scales for the % methylation and CpG BS-seq coverage tracks have been added to the figure. The same has been done for Figure 2D.*

“Figure 1E: is the example shown in a region of relatively low CpG coverage? The tracks in Figure 2D look more convincing - is the scale the same? Is the genomic region shown in Figure 1E and 2D actually the same (the genomic region window on chromosome 11 is certainly similar)?”.

*The peaks shown in Figures 1E and 2D were of the same region. The global BS-seq coverage is markedly lower for the PGC data than for the pre-implantation and gamete data, so indeed the image we showed originally in Figure 1E had lower coverage than Figure 2D. We now include a different example with relatively good BS-seq coverage in all the PGC and preimplantation samples and we now show this region in Figures 1E and 2D. As before, the peaks in Figures 1E and 2D are in the same region; we have made this more explicit in the Legend for Figure 2. In these new panels, in order to reduce noise, we only display BS-seq data at CpGs with at least 6-fold coverage on both strands combined in the PGC data and at least 10-fold coverage on both strands in the pre-implantation/gamete data. In the original version of the manuscript we displayed all CpGs in these panels regardless of coverage.*

“Figure 2E: no scale - is it the same as in Figure 1G?”.

*We have added a scale to the figure; it is the same as in Figure 1G.*

“Page 7, line 30-32: I suggest changing "…set of regions with low DNase-seq signal in E9.5 PGCs (E9.5-trace) but with high DNase-seq in E14.5 PGCs (E14.5m-Hi), and compared them to…" to: "…set of regions with low DNase-seq signal in E9.5 PGCs but with high DNase-seq in E14.5 PGCs (E9.5-trace, E14.5m-Hi), and compared them to…". A cosmetic change but makes it easier to follow Figure 3.”

*We changed the text as suggested by the reviewer. These sentences are now in page 9, lines 8-11.*

“Page 7, lines 40-41: "Both E9.5-Hi, E14.5m-trace and E9.5-trace, E14.5m-Hi show no discernible pattern of TF binding during pre-implantation development and in sperm (Figure 3C)." I tend to agree with this conclusion for E9.5-trace, E14.5m-Hi (upper panels), but there appear to be quite pronounced changes for E9.5-Hi, E14.5m-trace (lower panels)”. (now page 9, lines 17-19).

*We changed that sentence, “Both E9.5-Hi, E14.5m-trace and E9.5-trace, E14.5m-Hi sites…” to “TF binding in sperm and pre-implantation embryos occurs at some but not all of the E9.5-Hi, E14.5m-trace and E9.5-trace, E14.5m-Hi sites, and specific sites gain or lose TF occupancy at different stages in this time range.” We no longer state that no discernible pattern exists.*

“Figure 3C: are the numbers correct here, n=192 for the upper panels and n=984 for the lower panels? This is hard to verify for based on the figure for me but my impression is that more sites are shown in the upper panels compared to the lower panels?”. The numbers are correct but agree with the reviewer that the way the information was presented was confusing.

*The two heatmaps were not drawn to scale, because if they were, readers would be unable to see the E9.5-trace, E14.5m-Hi set in detail, since it is only one tenth the size of the E9.5-Hi, E14.5m-trace set. In order to ensure that readers are not confused by this, we have added an explicit statement in the figure legend that the scales are different.*

“Figure 4E: are both mouse and human TFs listed here? If yes, why (as dynamic methylation of the mouse genome during development is the focus of this study)? If not, why is the nomenclature not consistent (for example HOXD13 but Hoxd12)?”.

*We used both human and mouse motifs because the database of mouse motifs is smaller than that for humans, and therefore including human motifs enabled us to increase the information about TF binding at sites with DNAse signal. This is the default behavior when using the online version of fimo. However, we only used human motifs for TFs where a mouse motif was not available. We have added a brief description of why we used both human and mouse motifs on page 6, lines 3-7.*

“Figure 5G legend: "Example showing the Nrf1 ChiP-seq (minor: should be ChIP-seq) and DNA methylation levels during and after growth in 2i medium at a Nrf1 peak with relatively high TRAP affinity." Is this correct? My interpretation of the figure is that two different Nrf1 binding sites are shown, presumably one with low TRAP affinity (gaining DNA methylation and losing Nrf1 binding) in the upper panels and a different one with high TRAP affinity (sustained Nrf1 binding and no changes in DNA me) in the lower panels?”.

*We have now changed “ChiP-seq” to “ChIP-seq”. Also, the reviewer is correct in pointing out that there are two separate Nrf1 binding sites shown in the panel, one with low and the other with high binding affinity. We have changed the description in the figure legend accordingly. Page 7, line 40-41 (now page 9, lines 17-19): We changed the sentence, “Both E9.5-Hi, E14.5m-trace and E9.5-trace, E14.5m-Hi sites…” to “TF binding in sperm and pre-implantation embryos occurs at some but not all of the E9.5-Hi, E14.5m-trace and E9.5-trace, E14.5m-Hi sites, and specific sites gain or lose TF occupancy at different stages in this time range.”*

“Several typos in the discussion on page 13:

- Line 34: "…miRNA's…" (should be miRNAs?)

- Lines 37/38: "…in pre-implantation embryos in studies in studies…"

- Lines 38/39: "…in a low protein diet-induced trans-generational epiphenotypes." Omit 'a'?

- Lines 39/40: "These studies also observed that the level on several piRNAs were altered in sperm…" should be "…was altered" or "…levels…were altered"?”.

*We have made all typographical corrections as suggested.*

“The design of figure 7 should be improved:

- Why are there three colours for CpG sites? Green for methylated, light blue for unmethylated and white for?

- The symbol for DNA demethylase should be consistent either a grey or a green diamond

- IAPs should be represented as a box with dashed lines in the legend

- '2Cell morula' is tilted”.

*We fully agree that the cartoons presented in Figure 7 to explain the model were poorly drawn and not very intuitive. We have now made the colors of CpGs and other symbols consistent throughout the figure. The prior inconsistency was an artifact of previous edits to the figure which were not noticed by the first author due to color vision impairments. Also, as per Reviewer 1’s comments, we have simplified the model substantially. Unnecessary symbols and labels were removed for simplification. Both the IAP symbol and “2Cell-morula” labels were removed.*

**Second round of review**

**Reviewer 1**

The authors have made a good attempt to answer many of the questions I had on the first submission. The paper proposes that transcription factors (TFs) protect DNA re-methylation in primordial germ cells and during preimplantation development. Furthermore, they propose that this could explain transgenerational epigenetic inheritance in mammals, which has yet to be fully explained.

Their idea is novel and perhaps the readers may be interested in debating and testing if there is strong evidence to support this proposition. I note that the authors have however had to correct some of their previous statements. For example on my point 2 on the involvement of TFs a key aspect of their hypothesis, the authors concede that persistence of the same TF may not be correct, and the actual factors bound might change. We do not have actual examples for this proposition, and it might be difficult to answer this question with certainty. They have also withdrawn the claim that piRNAs could contribute to DNA de-methylation resistance (point13). There are other such examples. Overall, the manuscript has undergone considerable revision, and some of the statements have been corrected and rectified as outlined in the rebuttal document. Hence the manuscript is more balanced and less likely to be challenged because of the inaccuracies that were present in the previous version. I am however not able to judge if in doing so the authors may have also inevitably weakened their hypothesis.

In conclusion, the authors propose a new possibility to explain transgenerational epigenetic inheritance in mammals. There might be a case for getting the authors’ views across to the interested readers to see if in due course there is evidence to support their claims. I am myself not able to give a clear verdict based on what is presented in this paper.

**Authors Response**

**Point-by-point responses to the reviewers’ comments:**

*In regards to the reviewer’s idea that we have weakened the conclusions of the paper, I don’t really agree. Initially, the manuscript was written under the assumption that transcription factors bind to DNA and sterically interfere with methylation. In response to the suggestions of the reviewer, we have now changed the model to recognize that transcription factors could also recruit enzymes involved in DNA methylation or histone modifications, which in turn could affect the remethylation process. I don’t think this weakens the conclusions of the manuscript.*
